# Twist2 is NFkB-responsive when p120-catenin is inactivated and EGFR is overexpressed in esophageal keratinocytes

**DOI:** 10.1038/s41598-020-75866-0

**Published:** 2020-11-02

**Authors:** Heather L. Lehman, Michal Kidacki, Douglas B. Stairs

**Affiliations:** 1 Department of Pathology, The Pennsylvania State University College of Medicine, 500 University Dr., Mail Code H083, Hershey, PA 17033 USA; 2 Department of Biology, Millersville University, Millersville, PA 17551 USA; 3 Department of Internal Medicine, Mercy Catholic Medical Center, Darby, PA 19023 USA

**Keywords:** Oesophageal cancer, Molecular biology

## Abstract

Esophageal squamous cell carcinoma (ESCC) is among the most aggressive and fatal cancer types. ESCC classically progresses rapidly and frequently causes mortality in four out of five patients within two years of diagnosis. Yet, little is known about the mechanisms that make ESCC so aggressive. In a previous study we demonstrated that p120-catenin (p120ctn) and EGFR, two genes associated with poor prognosis in ESCC, work together to cause invasion. Specifically, inactivation of p120ctn combined with overexpression of EGFR induces a signaling cascade that leads to hyperactivation of NFkB and a resultant aggressive cell type. The purpose of this present study was to identify targets that are responsive to NFkB when p120ctn and EGFR are modified. Using human esophageal keratinocytes, we have identified Twist2 as an NFkB-responsive gene. Interestingly, we found that when NFkB is hyperactivated in cells with EGFR overexpression and p120ctn inactivation, Twist2 is significantly upregulated. Inhibition of NFkB activity results in nearly complete loss of Twist2 expression, suggesting that this potential EMT-inducing gene, is a responsive target of NFkB. There exists a paucity of research on Twist2 in any cancer type; as such, these findings are important in ESCC as well as in other cancer types.

## Introduction

Esophageal squamous cell carcinoma (ESCC) is among the most aggressive and fatal of all cancer types. As the most common subtype of esophageal cancer worldwide, ESCC invades and metastasizes rapidly, but typically remains asymptomatic until it impinges on the esophageal lumen and causes dysphagia^1,2^. As a result, ESCC is often diagnosed at a late stage, causing poor quality of life and mortality in the vast majority of patients^1–3^. Yet despite its dire nature, ESCC is still an understudied disease and little progress has been made on understanding the molecular pathways and mechanisms at play that make ESCC so devastating.

p120-catenin (p120ctn; *CTNND1*) is a tumor suppressor gene that is important for the stabilization of E-cadherin^4,5^. Importantly, p120ctn expression is down-regulated and/or lost in up to 60% of ESCC patients^4,6^ and associated with poor survival in ESCC and a number of other cancer types^4–22^. In previous studies we demonstrated the importance of the cooperation between p120ctn and another clinically relevant protein in ESCC, the epidermal growth factor receptor (EGFR). EGFR overexpression is commonly found in ESCC patients (up to 90%) and is also associated with poor prognosis and depth of invasion^23^. While p120ctn and EGFR are important on their own, we previously demonstrated that only when down-regulation of p120ctn and overexpression of EGFR occur together does it result in an aggressive and invasive cell type that closely mimics ESCC^24^. Furthermore, our previous studies show that p120ctn down-regulation and EGFR overexpression occurs simultaneously in 67% of human ESCC samples, making this a clinically-relevant condition represented in a majority of ESCC samples^24^.

Given the lack of information surrounding the molecular mechanisms controlling invasion in ESCC, we sought to know how p120ctn and EGFR create an invasive phenotype. Interestingly, we discovered that p120ctn down-regulation with EGFR overexpression leads to hyperactivation of NFkB p65 (Nuclear Factor kappa-light-chain-enhancer of activated B cells) (NFkB)^25^. NFkB is a major transcription factor that is involved in ubiquitous cellular processes such as inflammation, immune responses, angiogenesis, cell growth and proliferation, invasion and metastasis^26–28^. As suggested in our previous study, NFkB appears to be a major regulator of invasion as a result of its hyperphosphorylation and activity induced by p120ctn and EGFR in esophageal epithelial cells^25^.

While the role of NFkB in other cancers has been outlined fairly extensively^28–33^, little is known about the role of this clinically relevant gene in ESCC. Prior to our implicating NFkB in invasion, its upregulation was shown to be associated with advanced clinical stage and lymph node metastasis in ESCC^34^. It has also been suggested that NFkB activation is associated with ESCC tumor radioresistance^35^, potentially regulated through protein tyrosine kinase 7^36^. Given the limited investigations into a role for NFkB in ESCC, even less is known about the manner in which NFkB may regulate ESCC invasion. In our present study we aimed to identify targets that show differential expression in an NFkB-responsive manner. Using genetically modified human esophageal squamous keratinocytes, EPC cells (both EPC1 and EPC2 cell lines that are independent of each other), to assess NFkB-dependent changes, our data suggest that Twist2 (Dermo-1) is an NFkB-responsive gene when p120ctn is down-regulated and EGFR is overexpressed.

## Results

### The frequency of spindle cell morphology increases in esophageal keratinocytes when p120ctn is inactivated and EGFR is overexpressed

**Figure 1:**
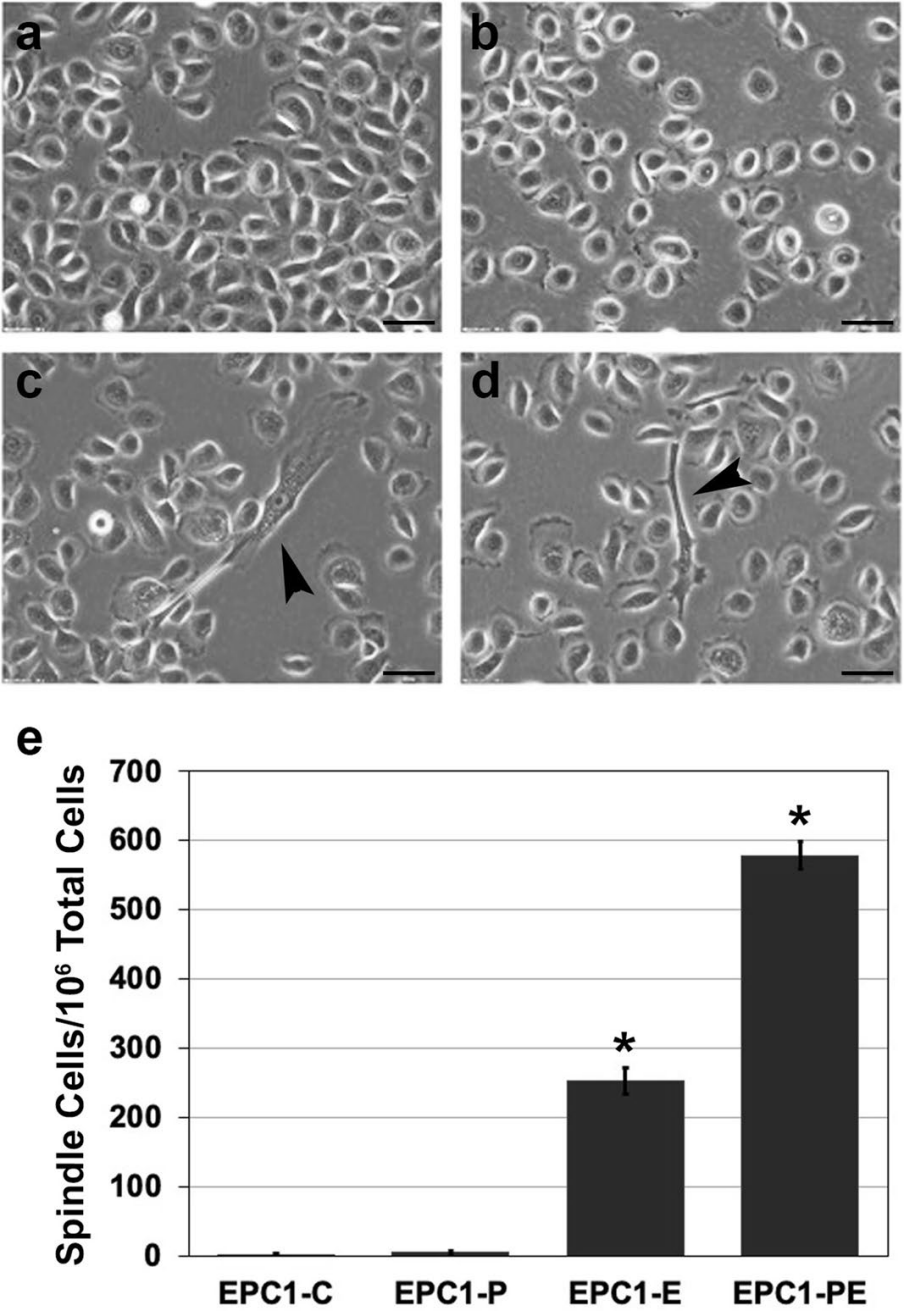
Pleomorphic/spindle cell morphology increases in cells with p120ctn inactivation and EGFR overexpression. (**a**) Phase contrast images of cell morphology demonstrate a rounded cell appearance of EPC1-C control cells grown in keratinocyte serum-free medium in 2D. (**b**) EPC1-P cells demonstrate a rounded appearance, though with less cell to cell contacts present. (**c**) EPC1-E cells demonstrate a growth pattern consistent with EPC1-C and EPC1-P cells, but with the occasional presence of a spindle-shaped cell. (**d**) When p120ctn inactivation is combined with EGFR overexpression in EPC1-PE cells, cell to cell adhesion is more inhibited and there is a significant increase in the number of pleomorphic/spindle shaped cells. (**e**) Quantification of pleomorphic/spindle shape EPC1 cells in 2D culture. *p < 0.05 for EPC-E versus EPC-C, EPC-PE versus EPC-C, and EPC-PE versus EPC-E. Arrows: spindle shaped cells.

When grown in keratinocyte serum-free media, the normal morphology of the epithelial cells that line the esophagus (esophageal keratinocytes) is expected to be fairly rounded. These round cells grow fairly close together, often in colonies (Fig. 1a). Inactivation of p120ctn in EPC1-P cells does not impact the rounded appearance of the cells, though cell to cell adhesion is decreased. This phenotype is expected, given the down-regulation of a p120ctn, a protein important for cell–cell adhesion (Fig. 1b). EGFR overexpression also does not greatly change the cell morphology, though an occasional EPC1-E cell is seen with a pleomorphic/spindle shape (Fig. 1c). Interestingly, EPC1-PE cells with combined p120ctn inactivation and EGFR overexpression have an increased number of cells with a pleomorphic/spindle shape, suggesting a number of cells possibly undergoing an epithelial to mesenchymal transition (EMT) (Fig. 1d). On average, it was found that approximately 250 out of every 1 million EPC1-E cells had a spindle shape. In the EPC1-PE cell population, there was a statistically significant increase in spindle-shaped cells, approximately 600 out of every 1 million. Quantification of the number of spindle-shaped cells present in a total population of esophageal keratinocytes is displayed in Fig. 1e (n = 3; *p* < 0.05). EMT is often not a complete effect, whereby there can be a range of epithelial and mesenchymal properties observed in a cell population^37–39^. Our data support this notion since not all of the cells in vitro demonstrate a strong shift in morphologic characteristics toward a mesenchymal/fibroblast appearance.

### p120ctn inactivation and EGFR overexpression results in increased Twist2 expression

Our previous studies have focused on the interaction between p120ctn and EGFR, two important genes in ESCC. We have demonstrated that the combination of p120ctn inactivation and EGFR overexpression is a state that is present in 67% of ESCC patients and is associated with poor prognosis^24^. ESCC is an understudied but aggressive disease, and since identifying p120ctn and EGFR as significant genes in ESCC, we have focused on investigating the mechanisms by which p120ctn and EGFR can cause invasion. Specifically, we demonstrated that p120ctn inactivation with EGFR overexpression causes hyperactivation of phosphorylated-NFkB, which is critical in the control of esophageal keratinocyte cellular invasion^25^.

It was a consistent observation in our cell cultures that many EPC1-PE cells had a pleomorphic/spindle shape in 2D culture; therefore, we took a candidate gene approach in this study to identify potential genes that may cause such a phenotype. We surveyed the literature in an unbiased manner, identifying multiple genes for further assessment based on their known and potential roles in cancer cell migration, invasion, and patient outcome. We selected four candidate genes to analyze potential changes in expression when p120ctn is down-regulated and EGFR is overexpressed – Integrin subunit alpha 1 (*ITGA1*)^40–43^, Laminin subunit alpha 4 (*LAMA4*)^44–46^, RhoC GTPase (*RHOC*)^47–51^, and Twist Family BHLH Transcription Factor 2 (*TWIST2*)^52–55^. The identified genes were also previously established to have interactions with EGFR.

**Figure 2:**
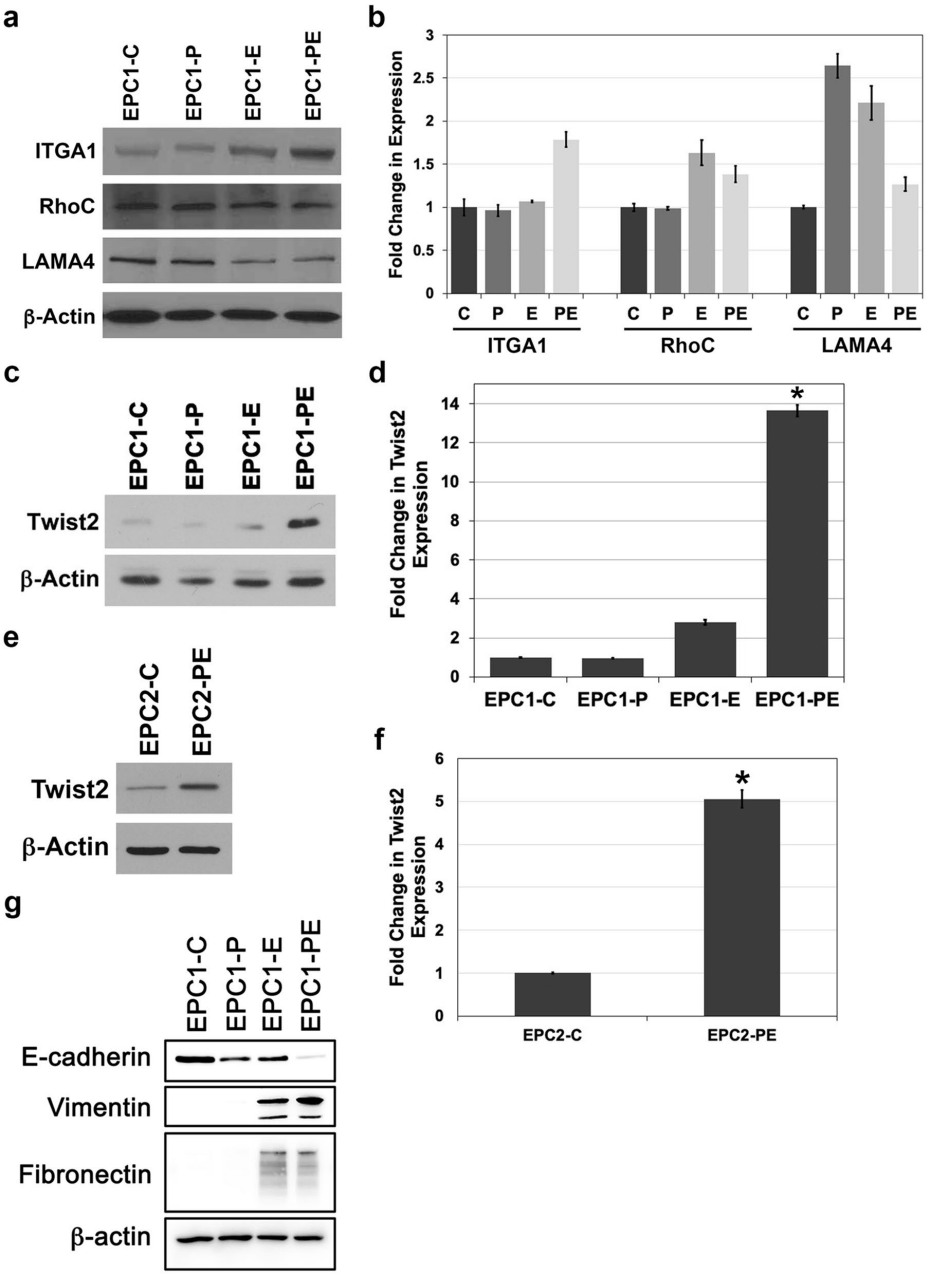
Twist2 expression levels are significantly increased in cells with p120ctn inactivation and EGFR overexpression. (**a**) Western blot analysis demonstrates protein expression of ITGA1, RhoC, and LAMA4 in EPC1 cells. Full-length blots are presented in Supplementary Figures S2–S5. (**b**) Quantification of ITGA1, RhoC, and LAMA4 expression, normalized to actin, in EPC1-C, -P, -E, and -PE cells. (**c**) Western blot analysis demonstrates a significant increase in Twist2 expression in EPC1-PE cells. Full-length blots are presented in Supplementary Figures S5–S7. Samples were derived from the same experiment and blots were processed in parallel. (**d**) Quantification of Twist2 expression, normalized to actin, in EPC1-C, -P, -E, and -PE cells. (**e**) Western blot analysis demonstrates a significant increase in Twist2 expression in EPC2-PE cells compared to control. Full-length blots are presented in Supplementary Figures S8–S9. Samples were derived from the same experiment and blots were processed in parallel. (**f**) Quantification of Twist2 expression, normalized to actin, in EPC2-C and EPC2-PE cells. (**g**) Western blot analysis demonstrates a decrease in E-cadherin in EPC1-P, EPC1-E and EPC1-PE cells, and an induction of Vimentin and Fibronectin in EPC1-E and EPC1-PE cells. Full-length blots are presented in Supplementary Figures S10–S13. *p < 0.05 for EPC-PE versus EPC-C, EPC-PE versus EPC-P, and EPC-PE versus EPC-E.

Upon qPCR analysis of our candidate genes, we detected a moderate two to threefold increase in mRNA expression of our four genes of interest in EPC1-PE cells (Table 1). Probing further into changes in protein expression, Western blot analysis demonstrated no significant change in expression of ITGA1, LAMA4, or RhoC (Fig. 2a). While we did observe a subtle 1.8-fold change in ITGA1 expression in EPC1-PE cells (Fig. 2b; n = 3), we saw a dramatic change in Twist2 expression levels in our cells when p120ctn and EGFR was modified compared to control cells (Fig. 2c). This observation led us to work further on investigating and quantifying Twist2 expression levels. On average, we saw a statistically significant 13-fold increase in Twist2 protein expression when both p120ctn is inactivated and EGFR is overexpressed compared to control cells with wild-type expression of p120ctn and EGFR (Fig. 2d; n = 5). Using an independent human esophageal keratinocyte cell line, EPC2 cells, we validated this increase in Twist2 protein expression induced by combined p120ctn inactivation and EGFR overexpression (Fig. 2e,f; n = 3).

**Table 1:** qPCR analysis of four candidate genes shows a modest increase in mRNA expression.

Gene	Fold change in mRNA expression compared to control
*ITGA1*	3.01
*LAMA4*	2.06
*RHOC*	3.12
*TWIST2*	2.97

Twist2 is a basic helix-loop-helix transcription factor family member that has long been known to be important during mammalian embryogenesis. More recently expression differences of Twist2 have been noted in a number of tumors, and importantly, Twist2 has been associated with advanced stages of disease and/or poor prognosis in a small number of squamous cancers^53,56–58^. Furthermore, increased expression of Twist2 has been shown to confer an EMT phenotype in a small number of cancers, primarily breast and ovarian^37,53,56–59^. However, nothing to date has been elucidated regarding any role for Twist2 in ESCC.

The phenotypic changes in our cells along with the significant up-regulation of Twist2 suggested to us that there could be an EMT shift occurring in part of our cell population(s). Therefore, we also chose to investigate a few classical markers of EMT (E-cadherin, Vimentin, and Fibronectin). E-cadherin is important in cell to cell adhesion and is therefore a marker of epithelial status. Conversely, vimentin and fibronectin are mesenchymal markers and indicative of a cell’s acquired ability to migrate and invade^60,61^. Western blot analysis of EPC1 cells demonstrates that E-cadherin is down-regulated in EPC1-P cells with p120ctn down-regulation. This is to be expected, as p120ctn is important in the stabilization of E-cadherin^4,62^. Furthermore, a slight decrease in E-cadherin is seen in EPC1-E cells with overexpressed EGFR. Interestingly, however, E-cadherin expression levels are inhibited even further in EPC1-PE cells (Fig. 2g; n = 3). Western blot analysis also demonstrates that the mesenchymal markers vimentin and fibronectin are induced in EPC1-E and EPC1-PE cells, with vimentin having the strongest increase in expression in EPC1-PE cells (Fig. 2g; n = 3). The down-regulation of E-cadherin and up-regulation of vimentin and fibronectin in EPC1-E and EPC1-PE cells, along with the observed phenotypic changes of the cells, indicate a potential EMT shift. Altogether, these data indicate that there is a slight or partial shift toward EMT in EPC1-E cells when EGFR is overexpressed alone. However, the EMT shift appears to be enhanced with the combination of p120ctn down-regulation and EGFR overexpression in EPC1-PE cells. As mentioned previously, Twist2 may also play a role in this process as has been shown in other cancer types. Indeed, the significant up-regulation of Twist2 in EPC-PE cells suggests the importance of investigating Twist2 further.

**Figure 3:**
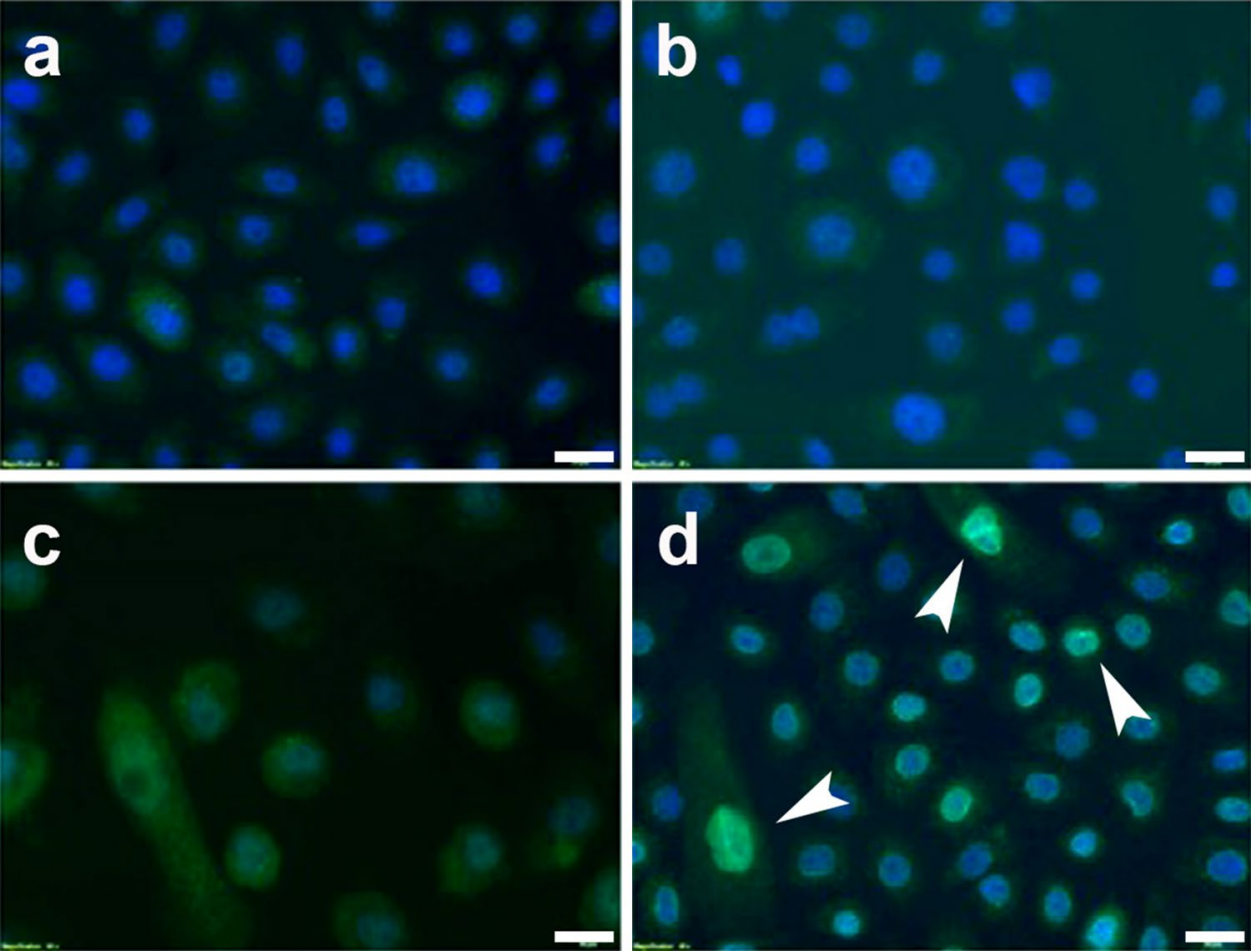
Nuclear expression of Twist2 is increased in esophageal keratinocytes with p120ctn inactivation and EGFR overexpression. Immunofluorescence staining of EPC1 cells demonstrates little Twist2 expression in EPC1-C (**a**) and EPC1-P cells (**b**). (**c**) A slight increase in Twist2 is seen in EPC1-E cells. (**d**) EPC1-PE cells show an increase in Twist2 expression, with a particular increase in nuclear expression noted in pleomorphic/spindle shape cells. Arrows: cells with high intensity nuclear staining. Scale bars = 50 uM.

We then further analyzed Twist2 expression in EPC1-PE cells by immunofluorescence (IF). IF analysis demonstrated an increase in Twist2 compared to control EPC1-C, -P, and -E cells (Fig. 3a–d; n = 3) As shown in Fig. 3d, the intensity of Twist2 staining in EPC1-PE cells is increased in the nucleus, particularly in pleomorphic/spindle-shaped cells. Collectively, these data demonstrate increased Twist2 expression at the mRNA and protein levels in esophageal keratinocytes with p120ctn inactivation and EGFR overexpression.

### Twist2 expression is increased in more invasive cells

**Figure 4:**
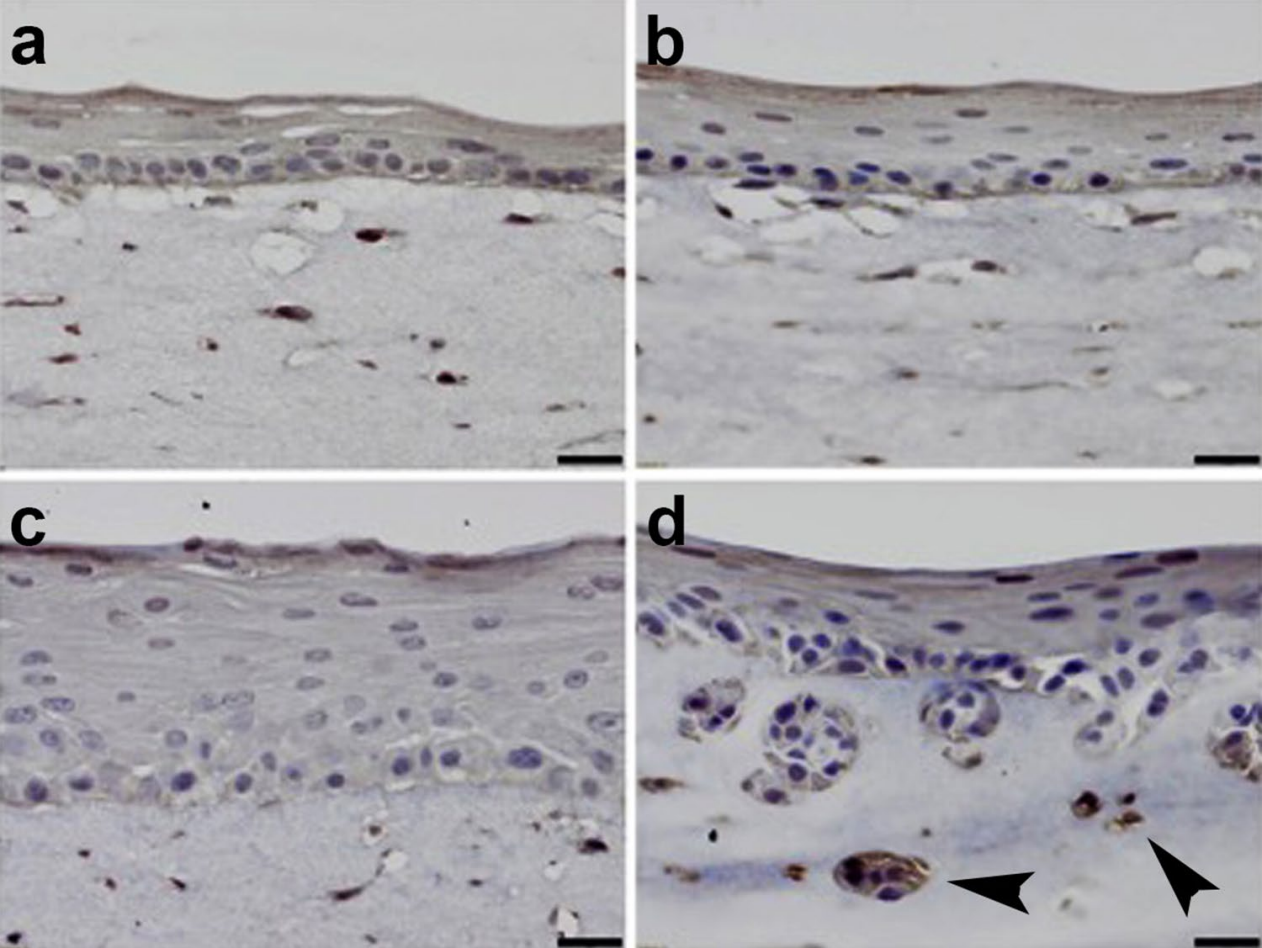
Twist2 expression increases in EPC1-PE 3D cultures. Immunohistochemical staining of (**a**) EPC1-C, (**b**) EPC1-P, and (**c**) EPC1-E 3D organotypic cultures demonstrates little Twist2 expression in the stratified squamous epithelium. (**d**) Twist2 is increased in 3D cultures with p120ctn inactivation and EGFR overexpression. Twist2 protein staining is most intense in cells that have deeply invaded in 3D culture. Arrows: deeply invaded cells with intense Twist2 staining. Scale bars = 50 uM.

Previous analysis of EPC1 and EPC2 esophageal keratinocytes in 3D organotypic culture, a model system that closely mimics the human esophageal epithelium^24^, demonstrated that p120ctn inactivation with EGFR overexpression induces a highly invasive phenotype. This is in contrast to 3D cultures with control cells or those that have only modification of p120ctn or EGFR^24^. IHC analyses of these cells in 3D culture demonstrate that EPC1-PE cells have increased Twist2 expression (Fig. 4a–d; n = 3). Additionally, Twist2 protein staining is the most intense in the most deeply invaded cells of the EPC1-PE organotypic culture (Fig. 4d, arrows). Interestingly, we have observed this staining pattern for pNFkB previously, where pNFkB staining intensity is the most robust in the most invasive EPC1-PE cells in 3D culture^25^.

### Inhibition of NFkB activity results in loss of Twist2 expression in EPC-PE cells

**Figure 5:**
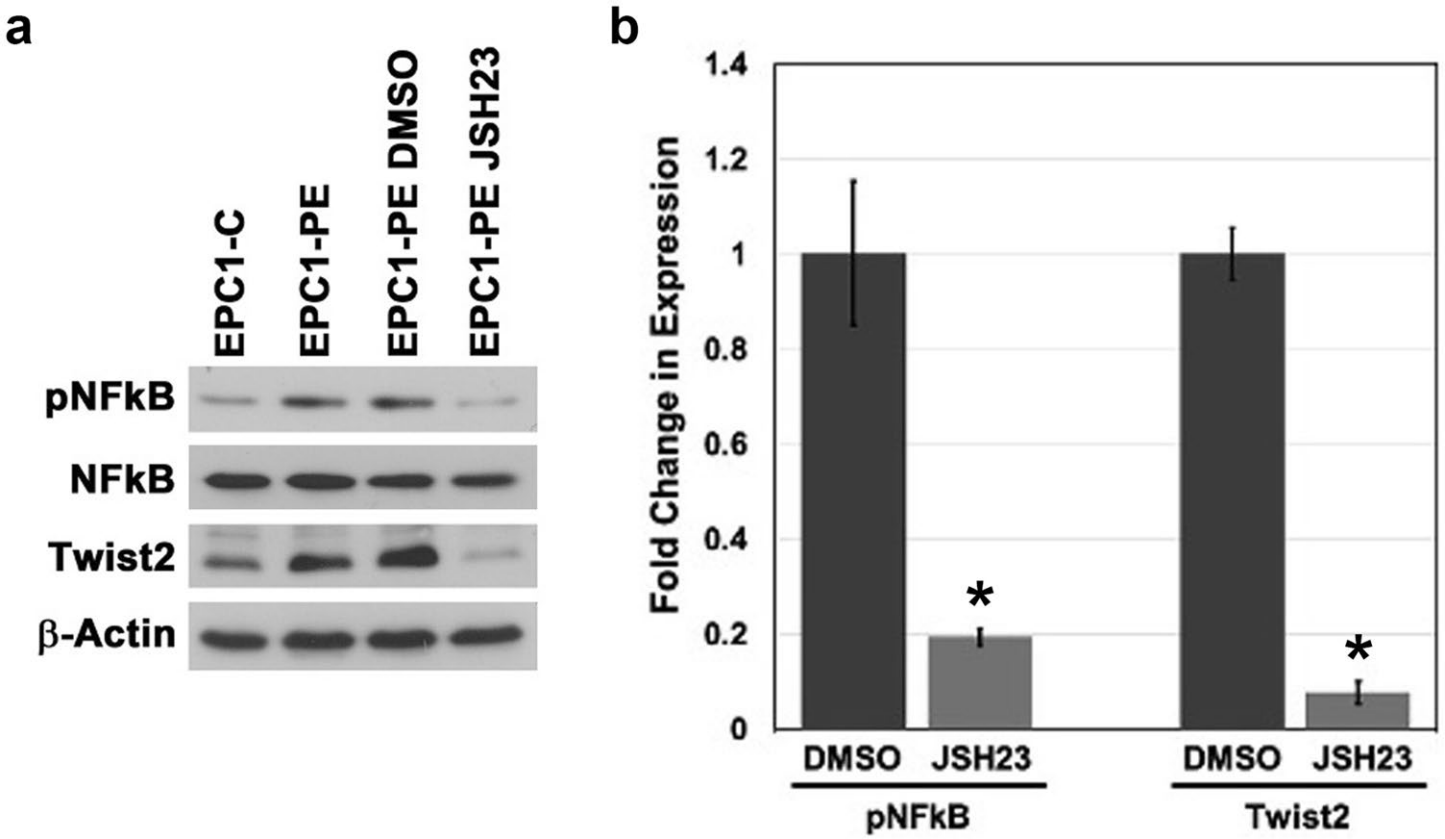
Twist2 is NFkB-responsive. (**a**) Western blot analysis demonstrates that inhibition of NFkB activity (pNFkB expression) with JSH-23 in EPC1-PE esophageal keratinocytes results in significant reduction in Twist2 expression. Full-length blots are presented in Supplementary Figure S14–S17. Samples were derived from the same experiment and blots were processed in parallel. (**b**) Quantification of pNFkB and Twist2 expression, normalized to actin, with JSH-23 treatment in EPC1-PE cells. *p < 0.05 for JSH23 versus DMSO.

Based on the similar expression pattern between NFkB and Twist2, we speculated that NFkB might transcriptionally regulate Twist2. To test this, we used JSH-23 to pharmacologically inhibit NFkB, as done in our previous studies (25). This compound selectively inhibits NFkB nuclear translocation, blocking NFkB activation and transcriptional activity^63^. Using this inhibitor, we inhibited activation of NFkB in EPC1-PE cells to levels below that seen in our control cells (Fig. 5a). We then examined Twist2 expression levels in cells with NFkB inhibition. Interestingly, Western blot analysis results demonstrated a statistically significant 13-fold decrease in Twist2 protein levels when NFkB activity is inhibited (Fig. 5a). Figure 5b displays the quantification of decrease in pNFkB levels and the resultant decrease in Twist2 expression (n = 4). To confirm these data, we also examined the effect on Twist2 protein expression by NFkB inhibition by JSH23 in EPC2 cells. Again, we see a robust decrease of Twist2 protein levels following NFkB inhibition (Supplementary Figure S1). These data suggest that Twist2 is responsive to activation levels of NFkB, a gene important in regulating invasion in an aggressive cancer type, ESCC, when p120ctn is inactivated and EGFR is overexpressed.

## Discussion

Esophageal tumorigenesis is a multistep disease process involving molecular and cellular mechanisms that rely on multiple genetic modifications. Growing evidence supports the overlap and integration of multiple molecular pathways that play key roles in the development and progression of cancer. Indeed, we were the first to demonstrate that p120ctn inactivation and EGFR overexpression intersect to cause an aggressive and invasive cell type that closely mimics human ESCC^24^. Not only does p120ctn and EGFR induce phenotypic and histologic changes that resemble ESCC, but modulation of these clinically relevant proteins leads to key molecular changes as well. Specifically, inactivation of p120ctn combined with overexpression of EGFR induces hyperactivation of NFkB p65 in human esophageal keratinocytes and ESCC^25^.

NFkB is a transcription factor that has been shown to regulate the expression of genes involved in many normal cellular processes as well as oncogenic processes, including proliferation, apoptosis, migration, and invasion. Our previous study delineated NFkB as a central regulator of invasion in ESCC^25^. We identified the signaling pathway that causes NFkB hyperactivation downstream of p120ctn inactivation and EGFR overexpression; however, the key downstream targets of NFkB that lead to an aggressive type of ESCC are unknown. In the present study, we have identified Twist2 as a gene that is responsive to NFkB activity. Specifically, we demonstrated high levels of Twist2 when NFkB hyperactivation is induced by modulation of p120ctn and EGFR together in human esophageal keratinocytes. Our data suggest that Twist2 expression is induced when NFkB activity rises above a certain threshold, since only when NFkB phosphorylation is very high in cells with both decreased p120ctn expression with EGFR overexpression do we see robust Twist2 expression. Moderate induction of NFkB phosphorylation by decreased p120ctn expression or EGFR overexpression individually does not induce significant Twist2 expression. To our knowledge, this present work is the first study identifying Twist2 as an NFkB-responsive gene.

Our initial observations of the unique spindle-shaped phenotype of human esophageal cells with p120ctn down-regulation and EGFR overexpression prompted our candidate gene approach to investigating genes that could potentially be responsible for such a phenotypic change. Twist2 is normally expressed in mesodermal tissues during embryogenesis and involved in differentiation of various cell lineages^64–67^. However, further studies have pointed to the oncogenic activation of Twist2 being important for the induction of epithelial-mesenchymal transition (EMT) in a few cancer types^37,53,56–59^. Twist2 has previously not been implicated in ESCC. During the EMT process, an epithelial cell undergoes multiple changes that allow it to transition to a mesenchymal cell that is capable of leaving the epithelial layer in which it originated. Some of these biochemical and phenotypic changes promote the acquisition of enhanced migration and invasion potential^37,68–73^, contributing to progressive tumor invasion, metastasis, and drug resistance^69–71,73^.

EMT is triggered by various signals, such as extracellular matrix and growth factors, cytoskeletal and cell-surface proteins, and activation of transcription factors^52,68,74–76^; one of potential importance being Twist2. However, it could be assumed that esophageal cells that have down-regulation of p120ctn are already primed for EMT given the inherent loss/down-regulation of E-cadherin in these cells^24^, which is a critical step and indicator of cells undergoing EMT. Furthermore, it has been suggested that EGFR may play a role in EMT by increasing matrix metalloproteinase-9^74^, activating STAT3 and Twist1^52^, or contributing to down-regulation of E-cadherin expression^75^. Certainly, as seen from our data, cells with inactivated p120ctn and overexpressed EGFR have an enhanced down-regulation of E-cadherin; even more so than cells that have inactivated p120ctn alone. With this in mind, we speculate that esophageal epithelial cells that have down-regulated p120ctn combined with overexpression of EGFR (our model system to study invasive ESCC) may be more prone to undergo the epithelial-mesenchymal transition. In support of this, the present study also demonstrated an induction of vimentin and fibronectin, markers of a cellular transition to a mesenchymal state and acquisition of an enhanced migratory and invasive potential. Indeed, we have clearly shown that EPC-PE cells have robust invasive capabilities^24,25^. Interestingly, as we have demonstrated in this study, these cells also have significantly increased Twist2 expression. Twist2 in these cells appears to be sensitive to NFkB activation, which we know to be crucial in the invasiveness of the cells^25^.

It is known that the invasive potential of cancer cells may eventually lead to metastasis and life-altering consequences. Therefore, the importance of understanding the underlying mechanisms that control invasion and EMT cannot be understated. The importance of NFkB in ESCC invasion is apparent, as our previous data demonstrate a significant increase in invasion with activation of NFkB and a complete loss of invasion with NFkB inhibition^25^. However, elucidating genes downstream of NFkB that may play a role in the aggressive nature of ESCC is critical. While NFkB may seem like a clear therapeutic target to slow the progression of ESCC, the challenges are numerous given that inhibition of NFkB, a required component of innate immunity, can lead to long-term immunosuppression and toxicity^25,76,77^. Therefore, there is an unmet need to find other potential molecular targets, including those downstream of NFkB. In order to advance research in this realm, there is a great need to investigate the role of Twist2 in ESCC. While we have established Twist2 as being responsive to NFkB when p120ctn is inactivated and EGFR is overexpressed, future studies will require understanding the functional consequences of Twist2 in cells with p120ctn and EGFR modulation. Specifically, it will be imperative to consider the possible role(s) that Twist2 is playing in EMT and invasion.

Overall, we have demonstrated that Twist2 expression increases significantly when p120ctn is down-regulated and EGFR is overexpressed. Twist2 is increased as a result of NFkB activation, as inhibiting NFkB results in a significant decrease in Twist2. Though further studies are warranted, Twist2 may be playing a role in causing ESCC tumor cells to undergo EMT and become more invasive.

## Materials and methods

### Cell lines

EPC-hTERT human esophageal keratinocytes, a gracious gift from Anil Rustgi, were cultivated in keratinocyte serum-free medium, as previously described^25,78^. Modifications of EPC cells were made with TRIPZ and pLVX vectors (Open Biosystems) to down-regulate p120ctn and overexpress EGFR, as previously described^24^. All derivatives of parental cells will be termed EPC-(genetic modification). EPC-C keratinocytes were generated by infection of parental EPC-hTERT cells with a pLVX-IRES-Neo vector and a TRIPZ lentiviral scrambled shRNA, resulting in no change in expression of p120ctn or EGFR. As previously described, EPC-P cells have down-regulated p120ctn expression, EPC-E cells have overexpression of EGFR, and EPC-PE cells have both p120ctn down-regulation and EGFR overexpression^24^. As previously described, human fetal esophageal fibroblasts (FEF-SC) were purchased from ScienCell Research Laboratories, Inc. and were grown in Dulbecco’s modified Eagle’s medium (DMEM) (Mediatech, Inc.) supplemented with 10% fetal bovine serum (FBS) (VWR) and 1% penicillin/streptomycin (Invitrogen)^25^. All cell lines were cultivated at 37 °C with 5% CO_2_.

Pleomorphic/spindle-shaped cells were quantified by counting the number of spindle-shaped cells per 1 × 10^6^ total cells. Averages were calculated across a minimum of three replicates at a minimum of three cell passages.

### qPCR

EPC cells were cultivated, and plates were harvested when confluency reached 60%. After washing cells with ice-cold Phosphate Buffered Saline, total RNA was isolated using Trizol extraction following the steps outlined by the manufacturer (Invitrogen). Twist2 mRNA expression was determined by qPCR using SYBR Green PCR master mix (Sigma-Aldrich) using the manufacturer’s protocol and 5 pmol of forward and reverse primers.

### Western blot analysis

Cells were harvested by trypsinization and centrifuged at 1,000 rpm for 5 min. Cell pellets were washed in 1X PBS and centrifuged at 2,000 rpm for 5 min. Cells were then incubated in lysis buffer as previously described^24^. Protein concentrations were determined using a Coomassie Protein Assay Kit (Pierce Biotechnology). Denatured proteins were resolved by SDS-PAGE and transferred to polyvinylidene difluoride (PVDF) membrane (Immobilon-P; Millipore). Membranes to be visualized using chemiluminescence and film were incubated overnight at 4 °C with primary antibodies diluted in a PBST solution with 3% milk^24,25^. Horseradish peroxidase (HRP)-conjugated secondary antibodies were placed on the membranes for 1 h at room temperature in a 3% milk-PBST solution. Proteins were visualized using ECL Prime chemiluminescence (GE Healthcare) as previously described^24^, or with a FluorChem R System (ProteinSimple) according to the manufacturer’s instructions. Membranes to be visualized using fluorescent imaging were incubated overnight at 4 °C with primary antibodies diluted in a PBST solution with 5% bovine serum albumin (BSA) (Fisher Scientific). Fluorochrome-conjugated secondary antibodies were placed on the membranes for 2 h at room temperature in the dark in a 3% BSA-PBST solution. After washing membranes with PBST and PBS, proteins were visualized using the Typhoon FLA 9000 fluorescence detection system (GE Healthcare).

### 3D organotypic culture

EPC cells were grown on a 3D matrix as previously described^24,79^ with the following modifications^25^. The collagen/Matrigel layer had 1.5 × 10^5^ human fetal esophageal fibroblasts (FEF-SC cells) embedded within it. On day 13, cultures were raised to an air–liquid interface and cultured for four days in Epidermalization III medium, with medium changed every other day. This medium is identical to Epidermalization I medium except that it does not contain progesterone and 2% unchelated newborn calf serum is added. Cultures were grown for a total of 17 days and harvested by fixing half of the culture in 10% neutral buffered formalin (Fisher Scientific) and paraffin-embedding. The epithelium was peeled from the other half of the culture and processed for protein collection^25^.

### Antibodies and inhibitors

The Twist2 antibody was purchased from Abcam (#ab66031) and used at 1:5000 for immunoblotting, 1:1000 for immunohistochemistry and 1:500 for immunofluorescence. Antibodies for NFkB (1:1000; #4764), pNFkB (1:1000; #3033), ITGA1 (2 μg/ml; #71,747), Vimentin (1:5000; #9855), and E-cadherin (1:1000; #3195) were purchased from Cell Signaling Technology. The LAMA4 (1:1000; ab205568) and Fibronectin (1:15,000; ab45688) antibodies were purchased from Abcam. The RhoC antibody, used at a 1:1000 dilution, was graciously shared by Dr. Kenneth van Golen (University of Delaware). The β-actin antibody (1:10,000; #A5316) was used as a loading control for immunoblotting and was purchased from Sigma-Aldrich Corp. AlexaFluor488 anti-rabbit or AlexaFluor 568 anti-mouse secondary antibodies were used for Western blots scanned with the Typhoon FLA 9000. An AlexaFluor488 anti-rabbit secondary antibody was used for immunofluorescence. Inhibition of NFkB was performed using NFkB Activation Inhibitor II, JSH-23 (#481,408; Millipore) at a concentration of 10 μM for 2 h.

### Immunohistochemistry

Immunohistochemical techniques were carried out as previously described^24,25^. Paraffin sections of 3D organotypic cultures were baked for one hour at 60 °C and deparaffinized in xylene. Sections were then rehydrated in sequential 100%, 95%, 70% ethanol and distilled water. Antigen unmasking was performed with the Retriever (Electron Microscopy Sciences) in 10 mM sodium citrate buffer, pH 6. Endogenous peroxidases were quenched in 3% hydrogen peroxide for 6 min prior to sections being incubated for 30 min with a blocking solution of 5% BSA in PBS. Sections were incubated with primary antibodies overnight at 4 °C in 5% BSA, followed by 2 h with ImmPRESS HRP reagent (Vector Laboratories). HRP activity was then detected using DAB substrate (Thermo Scientific). Sections were dehydrated in 70%, 95%, and 100% ethanol and xylene, and coverslipped with Permount (Fisher Scientific). Sections were then imaged and photographed with an Olympus BX53 light microscope (Olympus America)^24,25^.

### Immunofluorescence

Cells were grown to a density of 70% confluence on Nunc chamber slides (Thermo Scientific). Cells were fixed with 4% formaldehyde for 15 min at room temperature, followed by PBS washing. A permeabilization step was performed with 0.1% Triton-X 100 in PBS for 15 min. Cells were incubated with 5% BSA in PBS for 1 h, followed by incubation with primary antibody in 5% BSA-PBS overnight at 4 °C. Fluorochrome-conjugated secondary antibody in 5% BSA-PBS was added for 2 h at room temperature in the dark. After washing with PBS, slides were then coverslipped using VectaShield mounting medium with DAPI^24^.

### Statistical analysis

A two-tailed Student’s *t*-test was used for pairwise comparisons to determine significant differences between cell lines or treatments. Image J software (version 1.53) was used to quantify immunoblots. *p* ≤ 0.05 was considered statistically significant for all experiments. All experiments were completed with a minimum of three biological and technical replicates.

## Supplementary information

Supplementary Information
