# Supplementary material for: Twist2 is NFkB-responsive when p120-catenin is inactivated and EGFR is overexpressed in esophageal keratinocytes
Source: Sci Rep. 2020 Nov 2;10:18829. doi: 10.1038/s41598-020-75866-0 (PMC7608670; doi:10.1038/s41598-020-75866-0)
Supplement: Supplementary file 1 — Supplementary Information [file 41598_2020_75866_MOESM1_ESM.docx]

**Twist2 is NFkB-responsive when p120-catenin is inactivated and EGFR is overexpressed in esophageal keratinocytes**

Heather L. Lehman^2^, Michal Kidacki^3^, Douglas B. Stairs^1^

**Affiliation:**

^1^ Department of Pathology, The Pennsylvania State University College of Medicine, Hershey, PA 17033

^2^ Department of Biology, Millersville University, Millersville, PA 17551

^3^ Department of Internal Medicine, Mercy Catholic Medical Center, Darby PA 19023

**Corresponding Author:** Douglas B. Stairs, Ph.D., Department of Pathology, The Pennsylvania State University College of Medicine, 500 University Dr., Mail Code H083, Hershey, PA 17033. E-mail: [dbs18@psu.edu](mailto:dbs18@psu.edu)

**
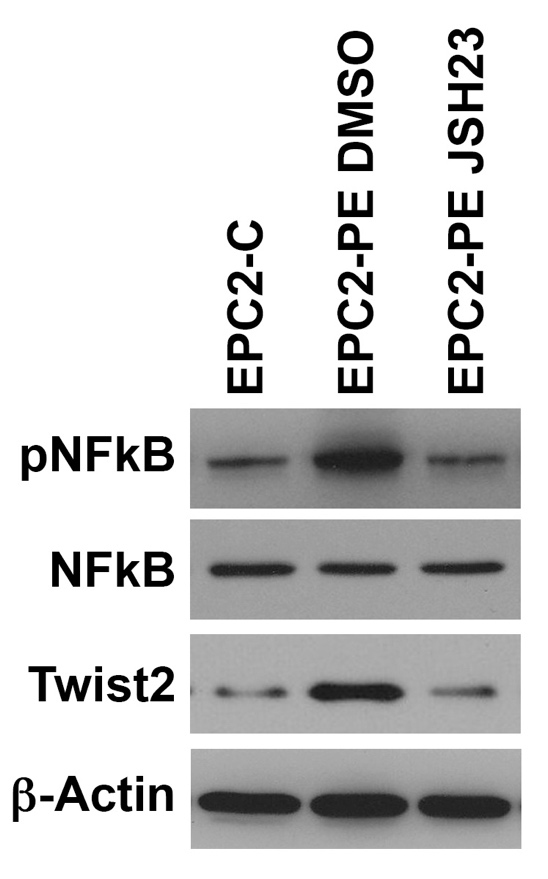
**

**Supplementary Figure S1.** Twist2 responds to NFkB in EPC-2 cells. Western blot analysis demonstrates that inhibition of NFkB activity (pNFkB expression) with JSH-23 in EPC2-PE esophageal keratinocytes results in a decrease in Twist2 expression. Full blots are presented in Supplementary Figures S18-S21. Samples were derived from the same experiment and blots were processed in parallel. (n=1)


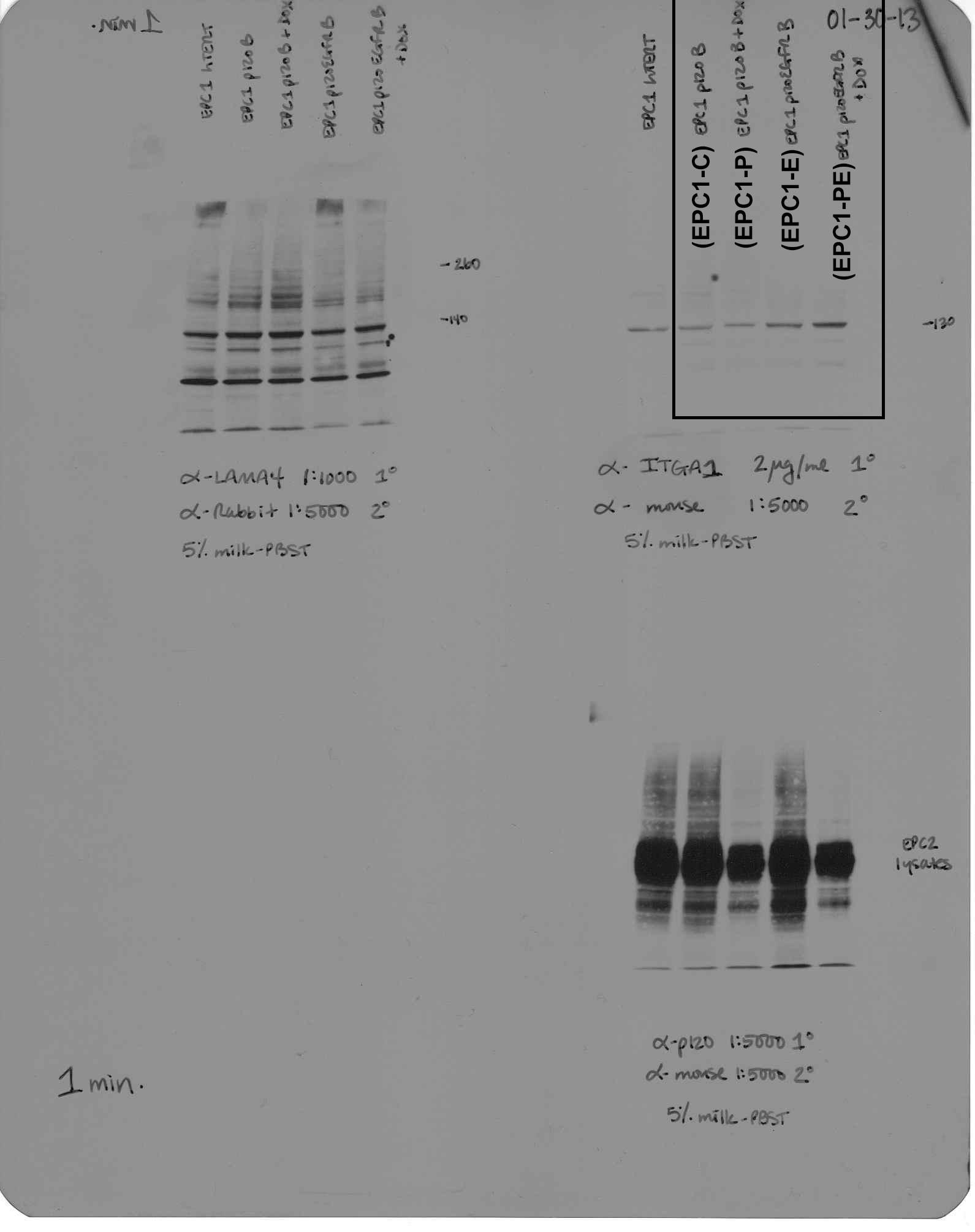


**Supplementary Figure S2.** Full-length Western blot of ITGA1 in EPC1 cells, visualized by chemiluminescence. Relevant experiment is marked with a black box and lanes have been labeled to match the main text. These data are presented in Figure 2a.


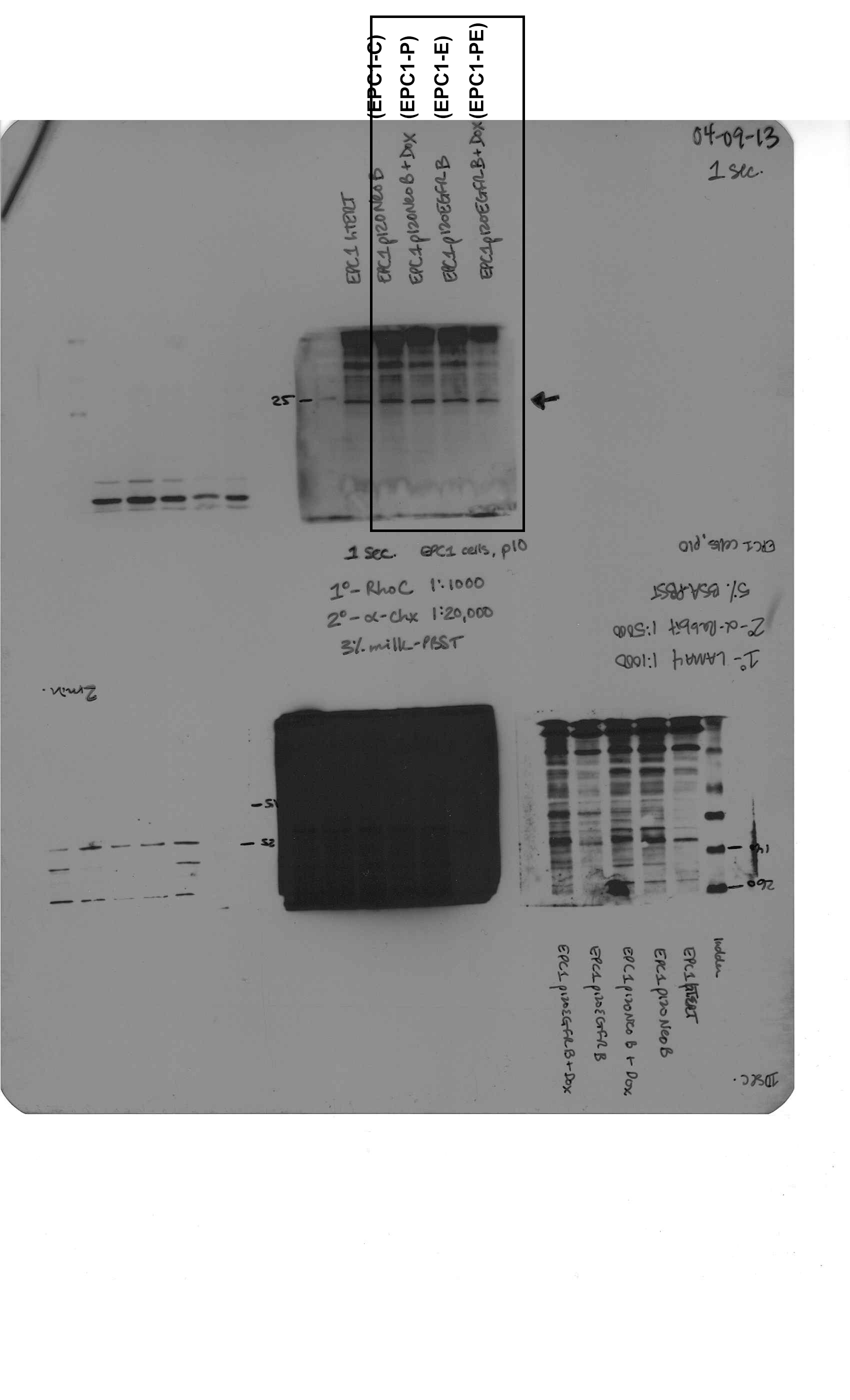


**Supplementary Figure S3.** Full-length Western blot of RhoC in EPC1 cells, visualized by chemiluminescence. Relevant experiment is marked with a black box and lanes have been labeled to match the main text. These data are presented in Figure 2a.


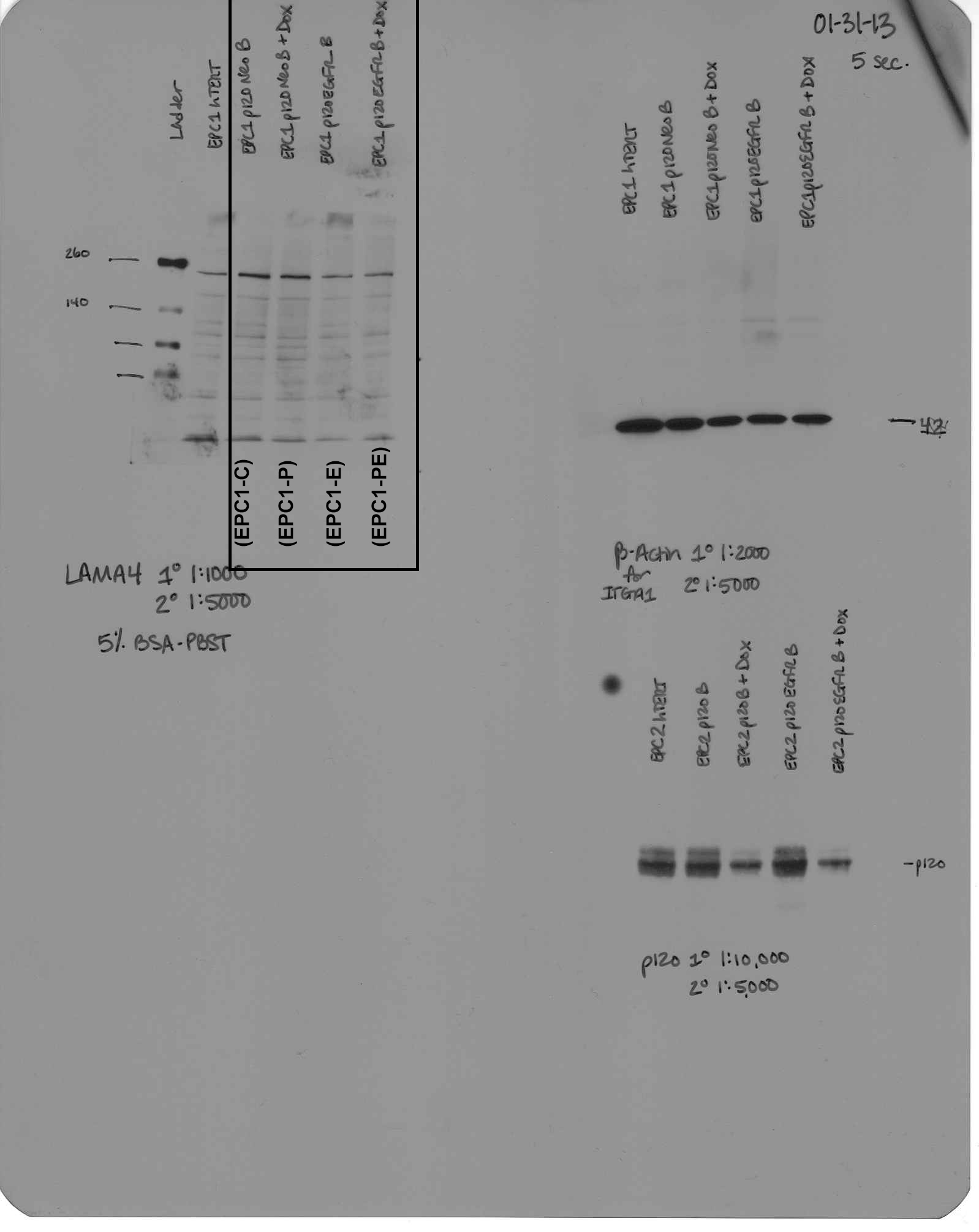


**Supplementary Figure S4.** Full-length Western blot of LAMA4 in EPC1 cells, visualized by chemiluminescence. Relevant experiment is marked with a black box and lanes have been labeled to match the main text. These data are presented in Figure 2a.


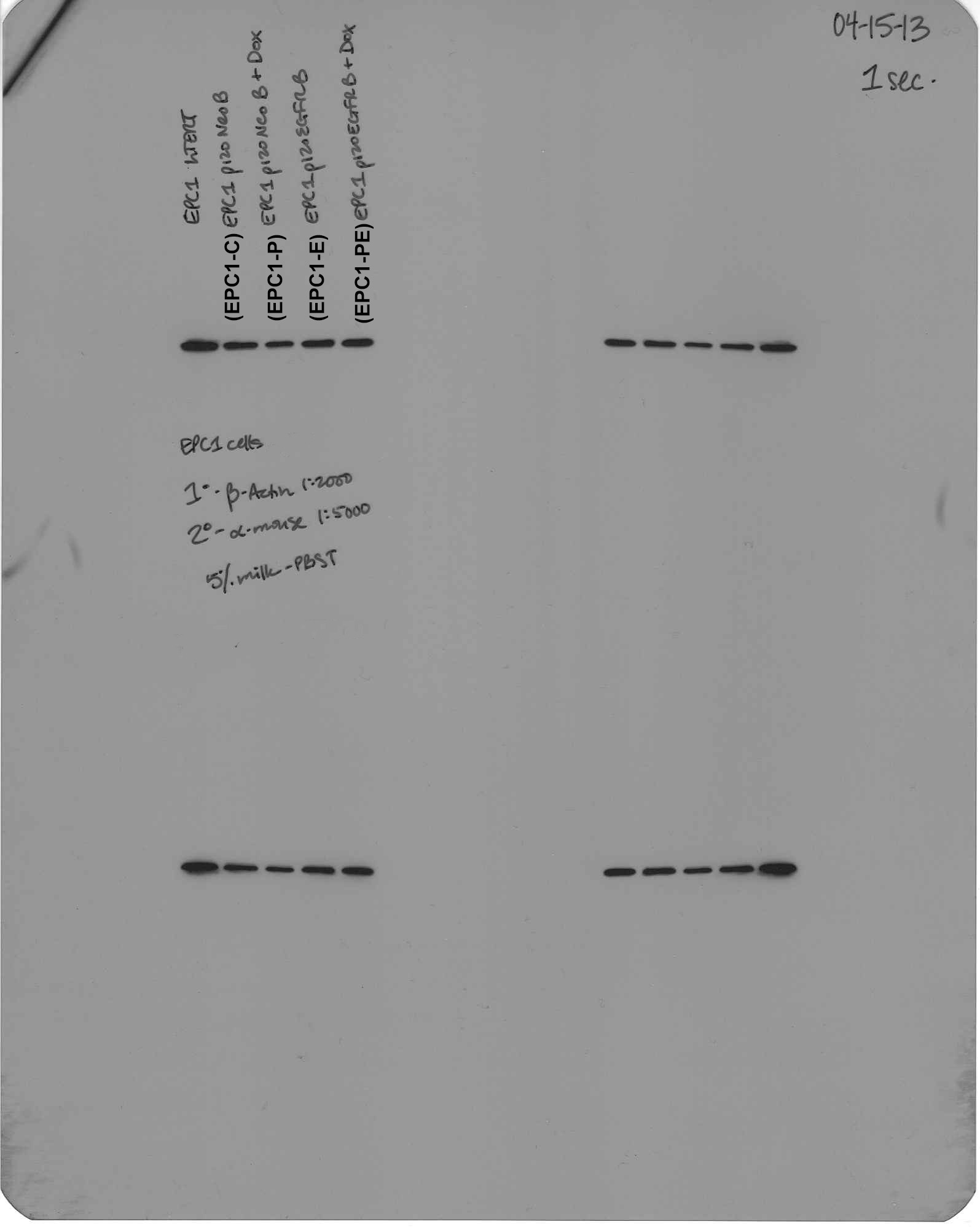


**Supplementary Figure S5.** Full-length Western blot of β-Actin, visualized by chemiluminescence. β-Actin was used as a loading control for ITGA1, RhoC, and LAMA4 in EPC1 cells. Relevant experiment is marked with a black box and lanes have been labeled to match the main text. These data are presented in Figure 2a.


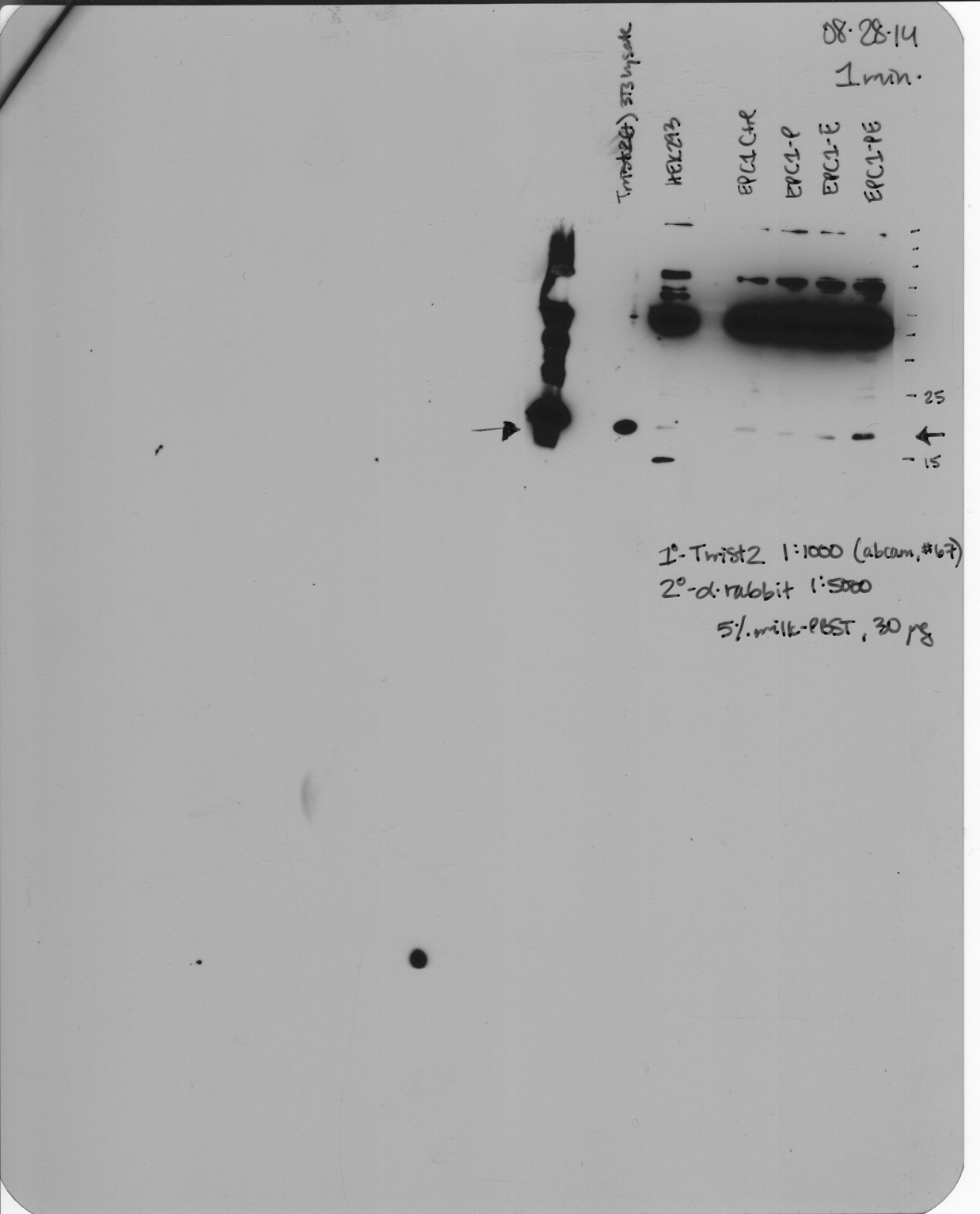


**Supplementary Figure S6.** Full-length Western blot of Twist2 in EPC1 cells, visualized by chemiluminescence. Relevant experiment is marked with a black box and lanes have been labeled to match the main text These data are presented in Figure 2c.


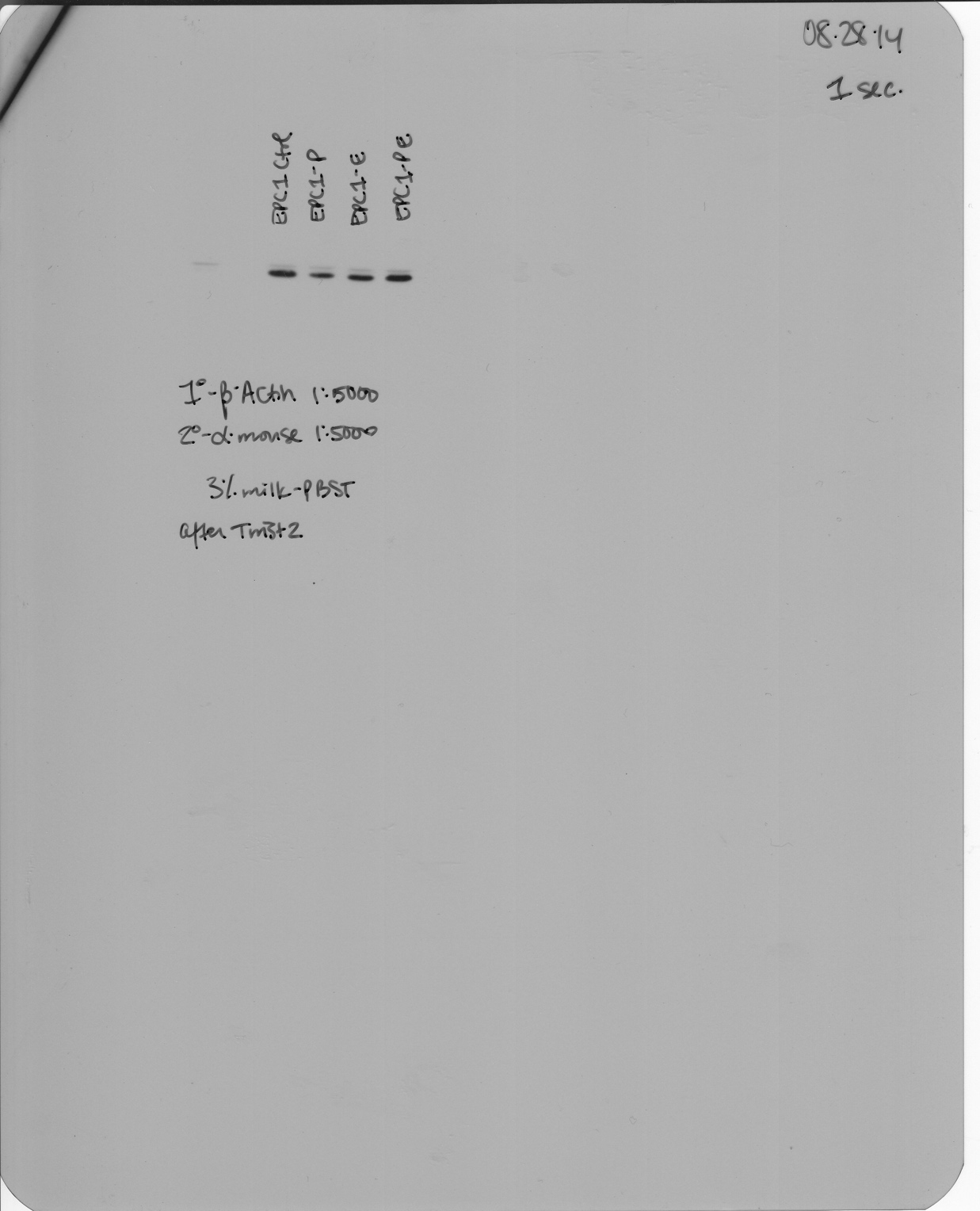


**Supplementary Figure S7.** Full-length Western blot of β-Actin, visualized by chemiluminescence. β-Actin was used as a loading control for Twist2 in EPC1 cells. These data are presented in Figure 2c.


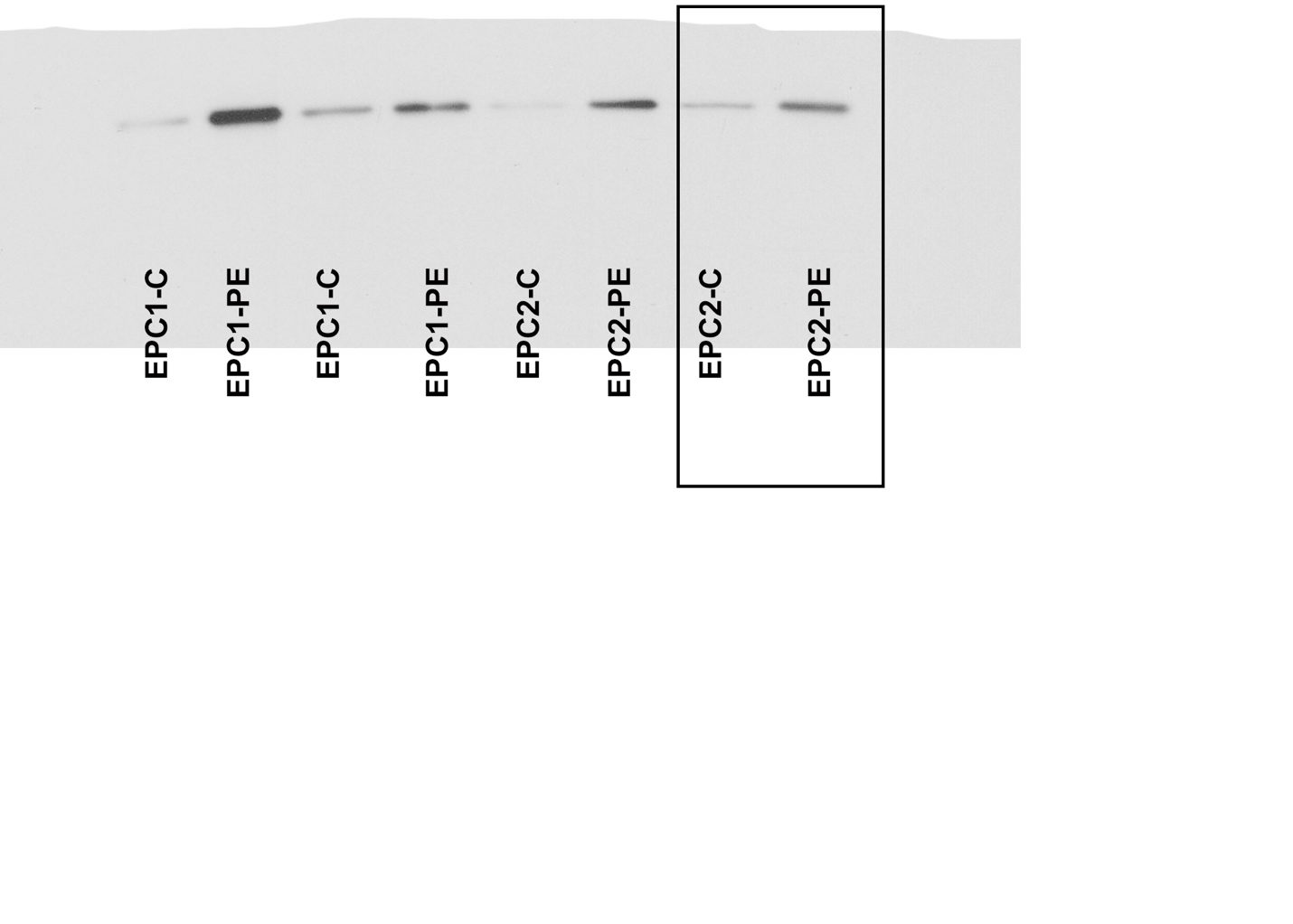


**Supplementary Figure S8.** Full-length Western blot of Twist2 in EPC2 cells, visualized by fluorescence using the Typhoon FLA 9000 system. Relevant experiment is marked with a black box and lanes have been labeled to match the main text. These data are presented in Figure 2e.


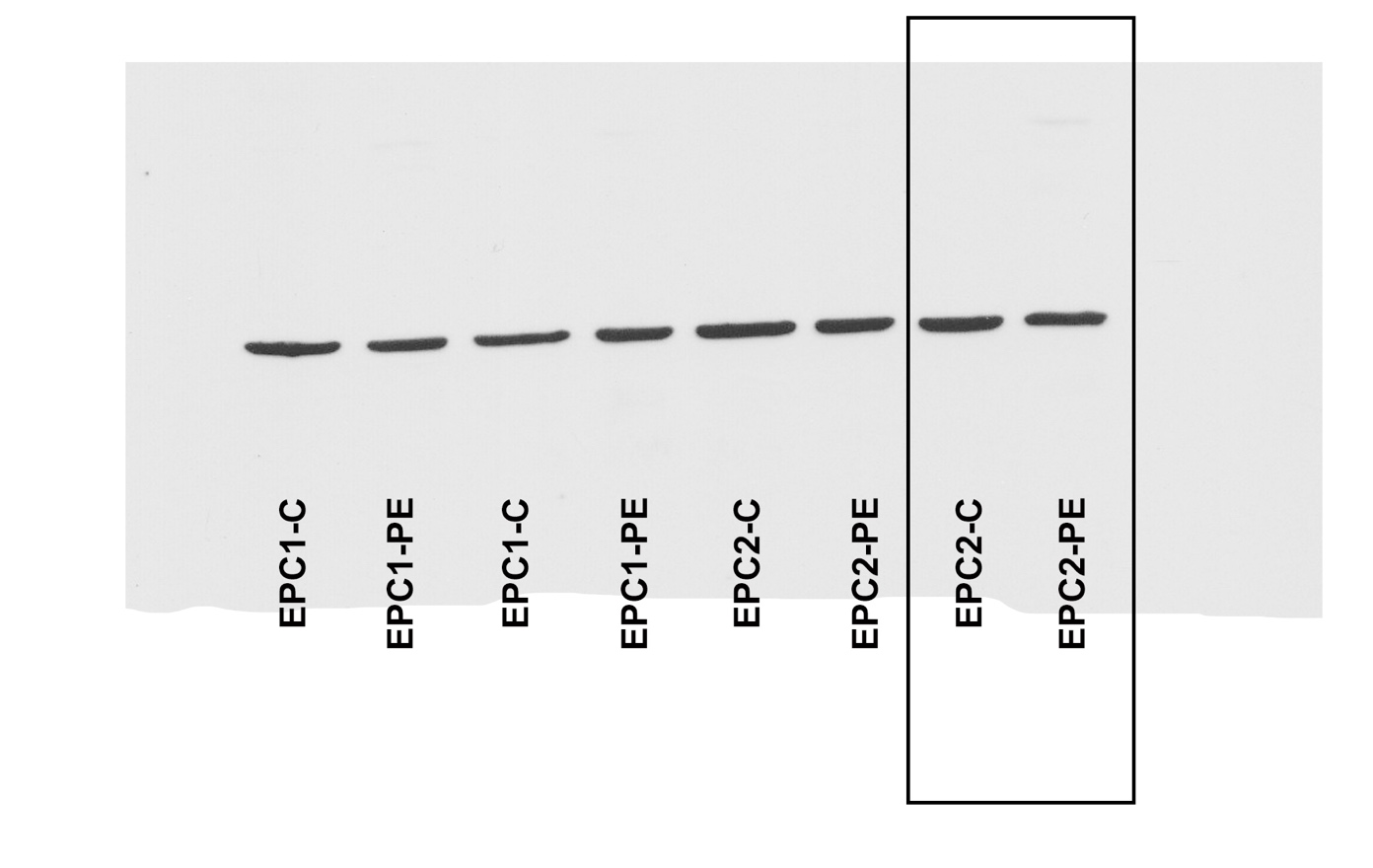


**Supplementary Figure S9.** Full-length Western blot of β-Actin in EPC2 cells, visualized by fluorescence using the Typhoon FLA 9000 system. β-Actin was used as a loading control for Twist2 in EPC2 cells. Relevant experiment is marked with a black box and lanes have been labeled to match the main text. These data are presented in Figure 2e.


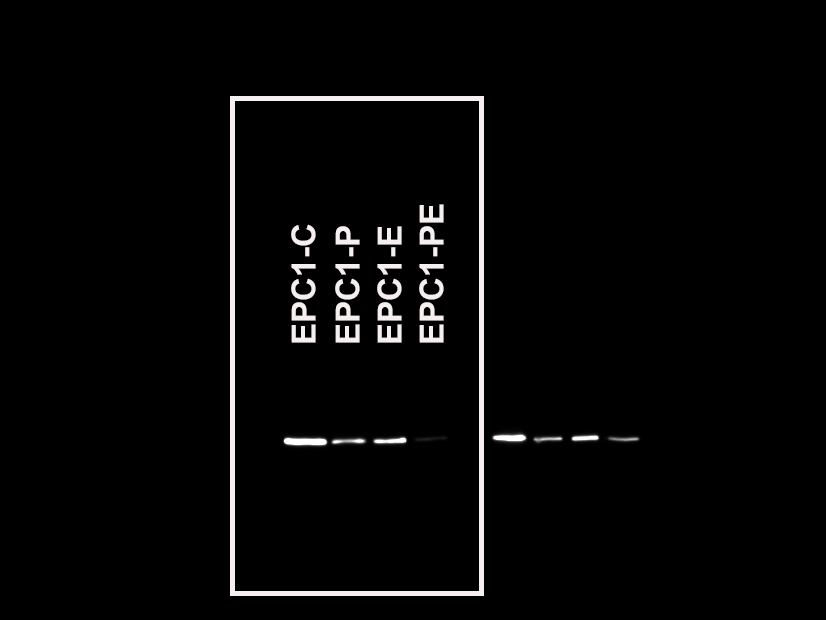


**Supplementary Figure S10.** Full-length Western blot of E-cadherin in EPC1 cells visualized by chemiluminescence using a ProteinSimple FluorChem R System. Relevant experiment is marked with a white box and lanes have been labeled to match the main text. For presentation in Figure 2g, this image was inverted to a white background to be consistent with other Western blot images presented; this was the only adjustment made to the image.


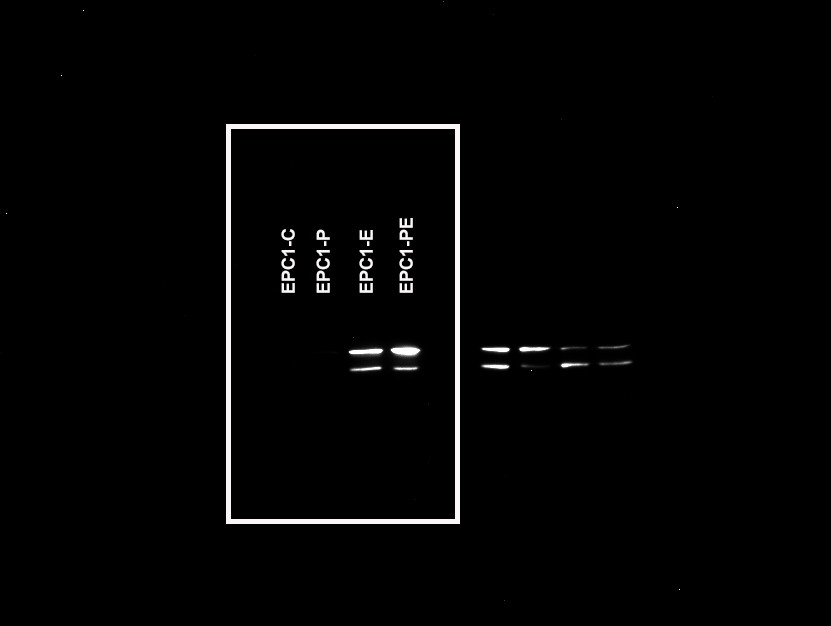


**Supplementary Figure S11.** Full-length Western blot of Vimentin in EPC1 cells visualized by chemiluminescence using a ProteinSimple FluorChem R System. Relevant experiment is marked with a white box and lanes have been labeled to match the main text. For presentation in Figure 2g, this image was inverted to a white background to be consistent with other Western blot images presented; this was the only adjustment made to the image.


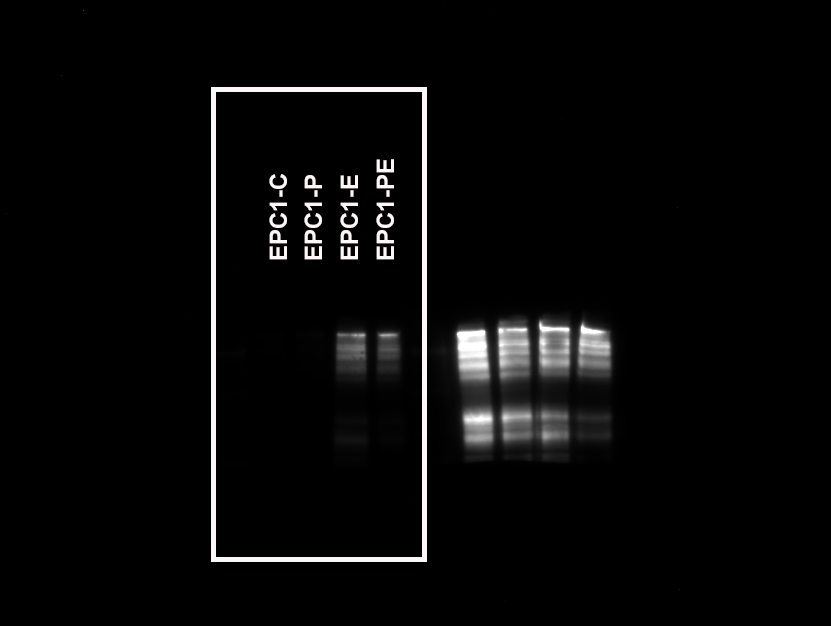


**Supplementary Figure S12.** Full-length Western blot of Fibronectin in EPC1 cells visualized by chemiluminescence using a ProteinSimple FluorChem R System. Relevant experiment is marked with a white box and lanes have been labeled to match the main text. For presentation in Figure 2g, this image was inverted to a white background to be consistent with other Western blot images presented; this was the only adjustment made to the image.


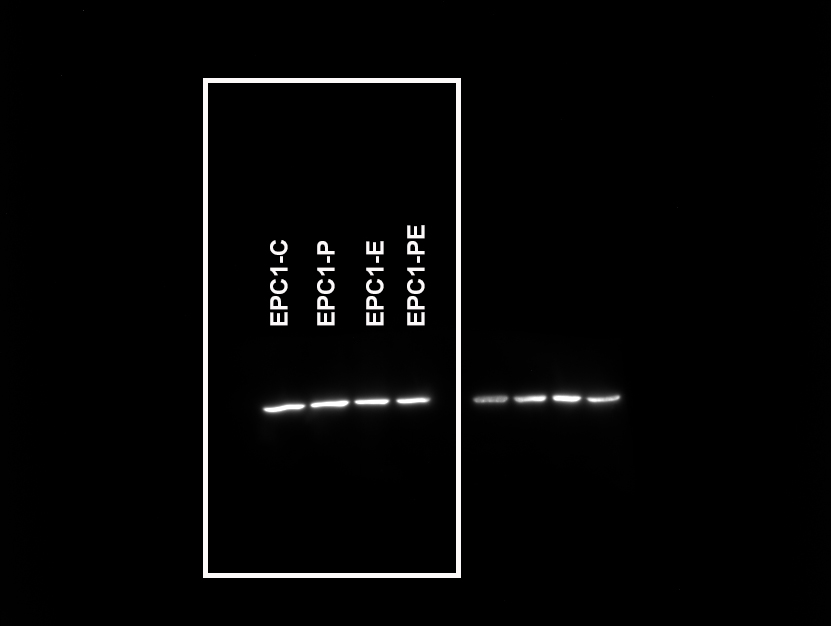


**Supplementary Figure S13.** Full-length Western blot of β-actin in EPC1 cells visualized by chemiluminescence using a ProteinSimple FluorChem R System. β-Actin was used as a loading control for E-cadherin, Vimentin, and Fibronectin in EPC1 cells. Relevant experiment is marked with a white box and lanes have been labeled to match the main text. For presentation in Figure 2g, this image was inverted to a white background to be consistent with other Western blot images presented; this was the only adjustment made to the image.


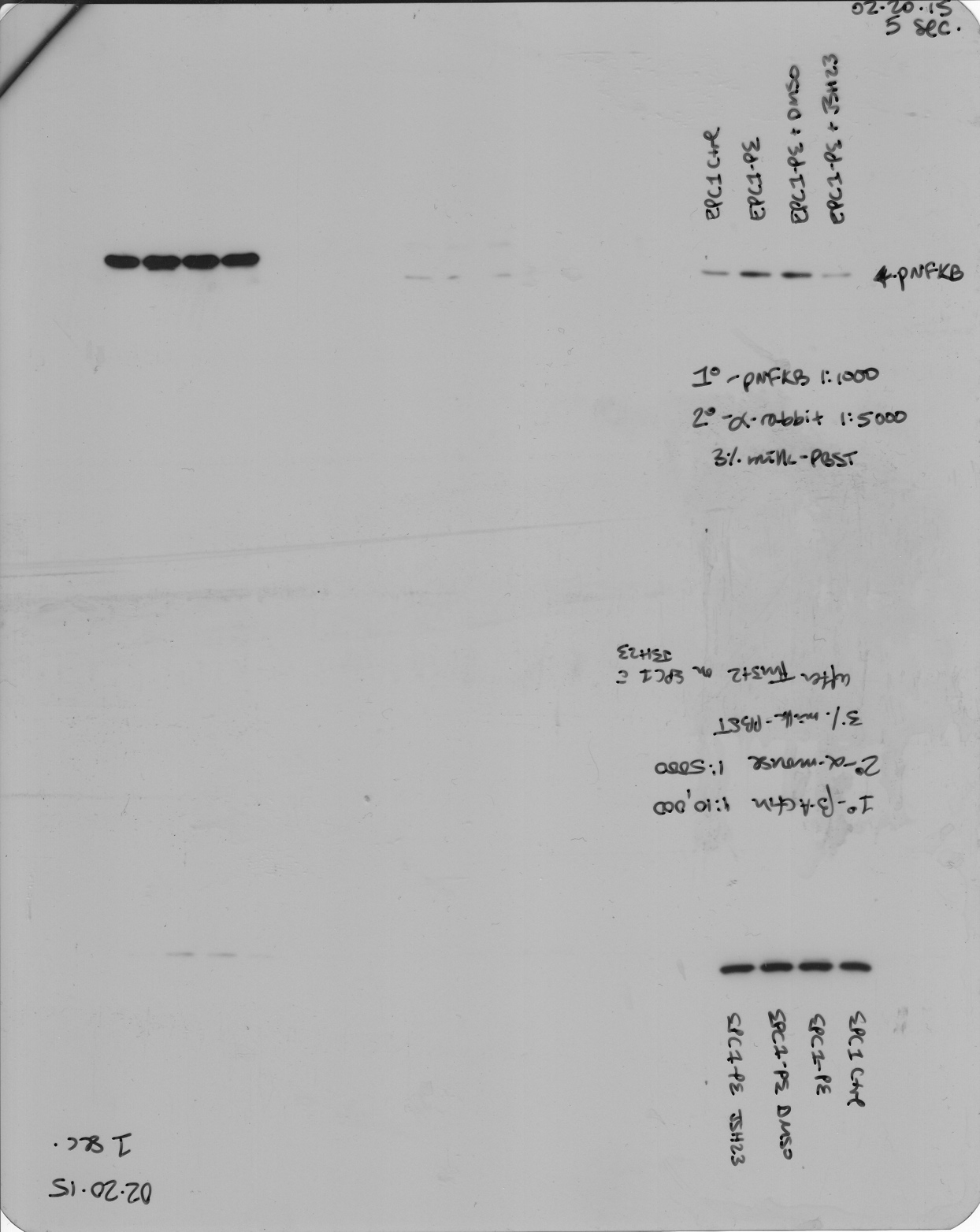


**Supplementary Figure S14.** Full-length Western blot of pNFkB in EPC1 cells treated with JSH23, visualized by chemiluminescence. Relevant component of the experiment is marked with a black box. These data are presented in Figure 5a.

.


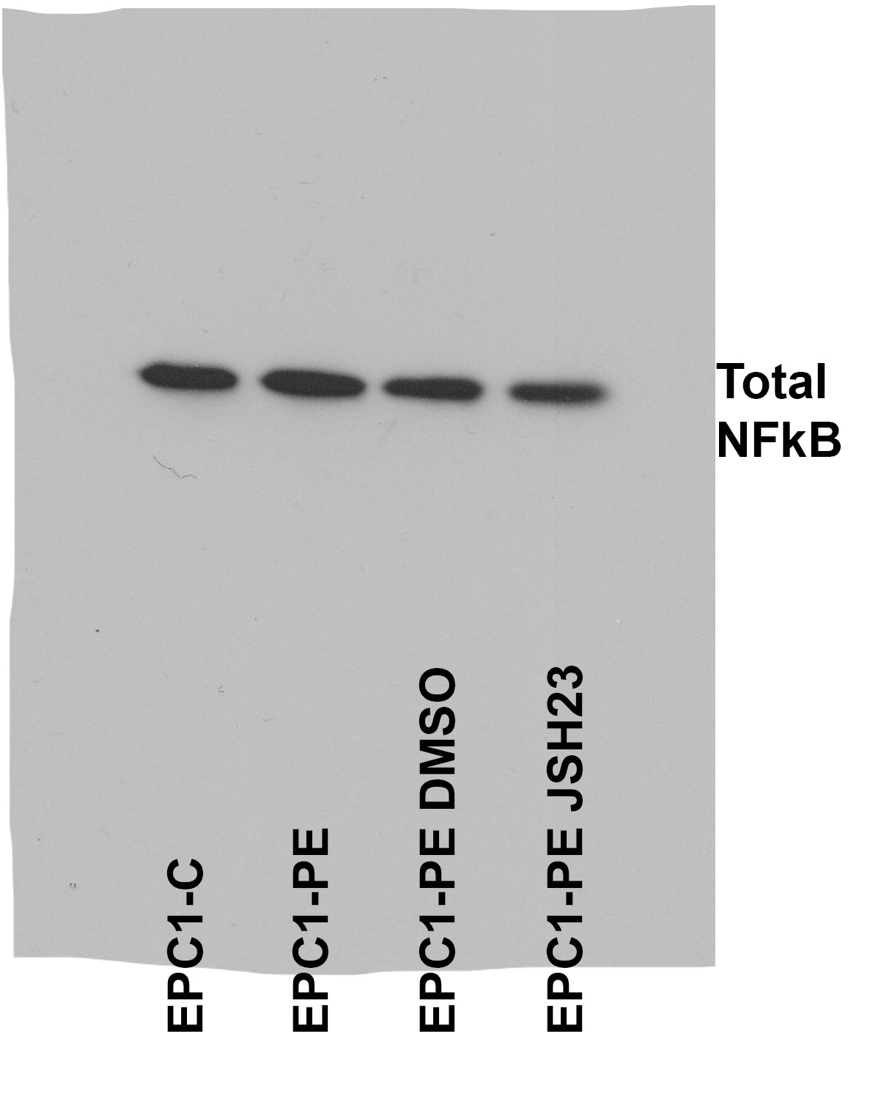


**Supplementary Figure S15.** Full-length Western blot of total NFkB in EPC1 cells treated with JSH23, visualized by fluorescence using the Typhoon FLA 9000 system. Relevant experiment is labeled to match the main text. These data are presented in Figure 5a.


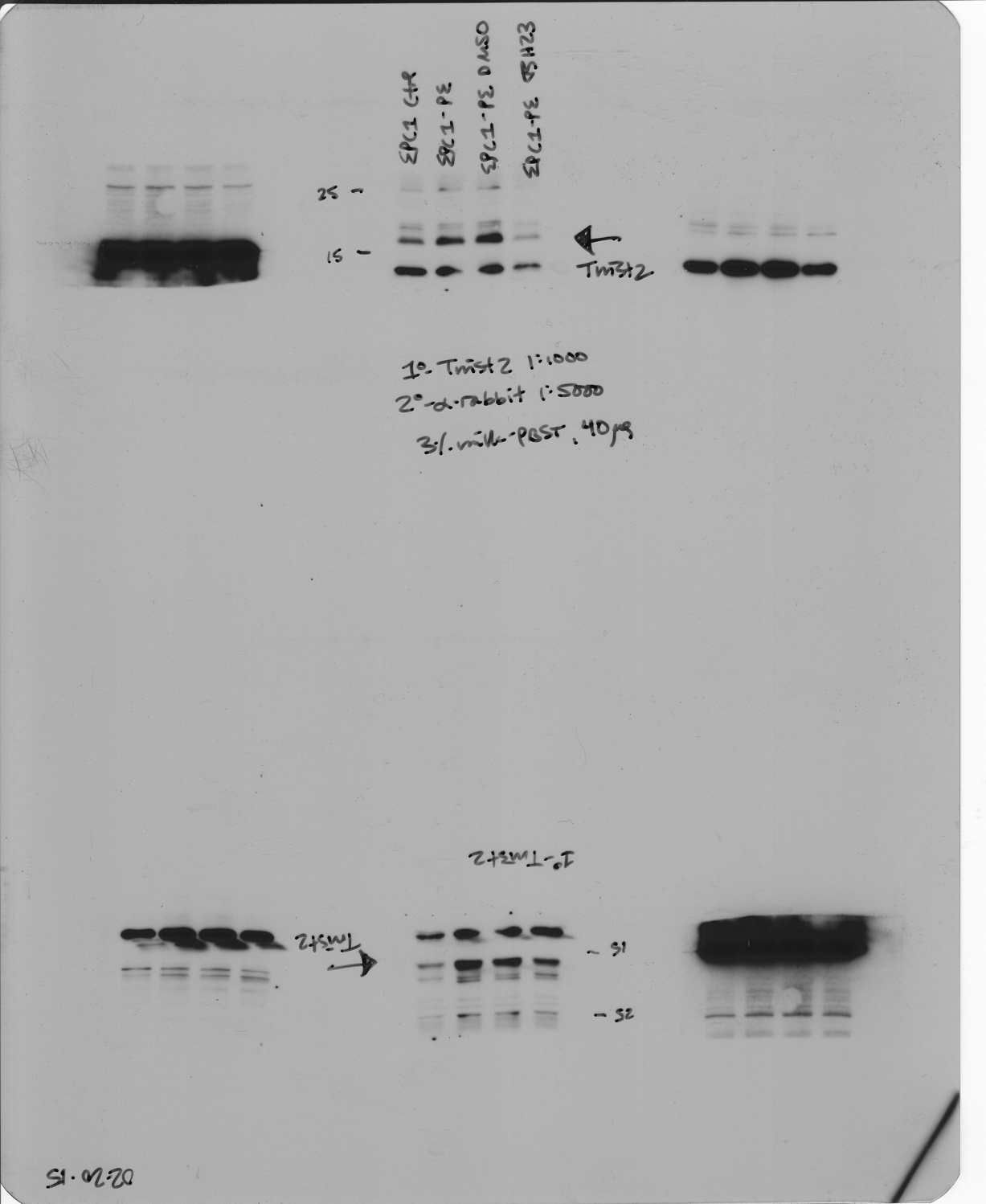


**Supplementary Figure S16.** Full-length Western blot of Twist2 in EPC1 cells treated with JSH23, visualized by chemiluminescence. Relevant experiment is marked with a black box and lanes have been labeled to match the main text. These data are presented in Figure 5a.

**
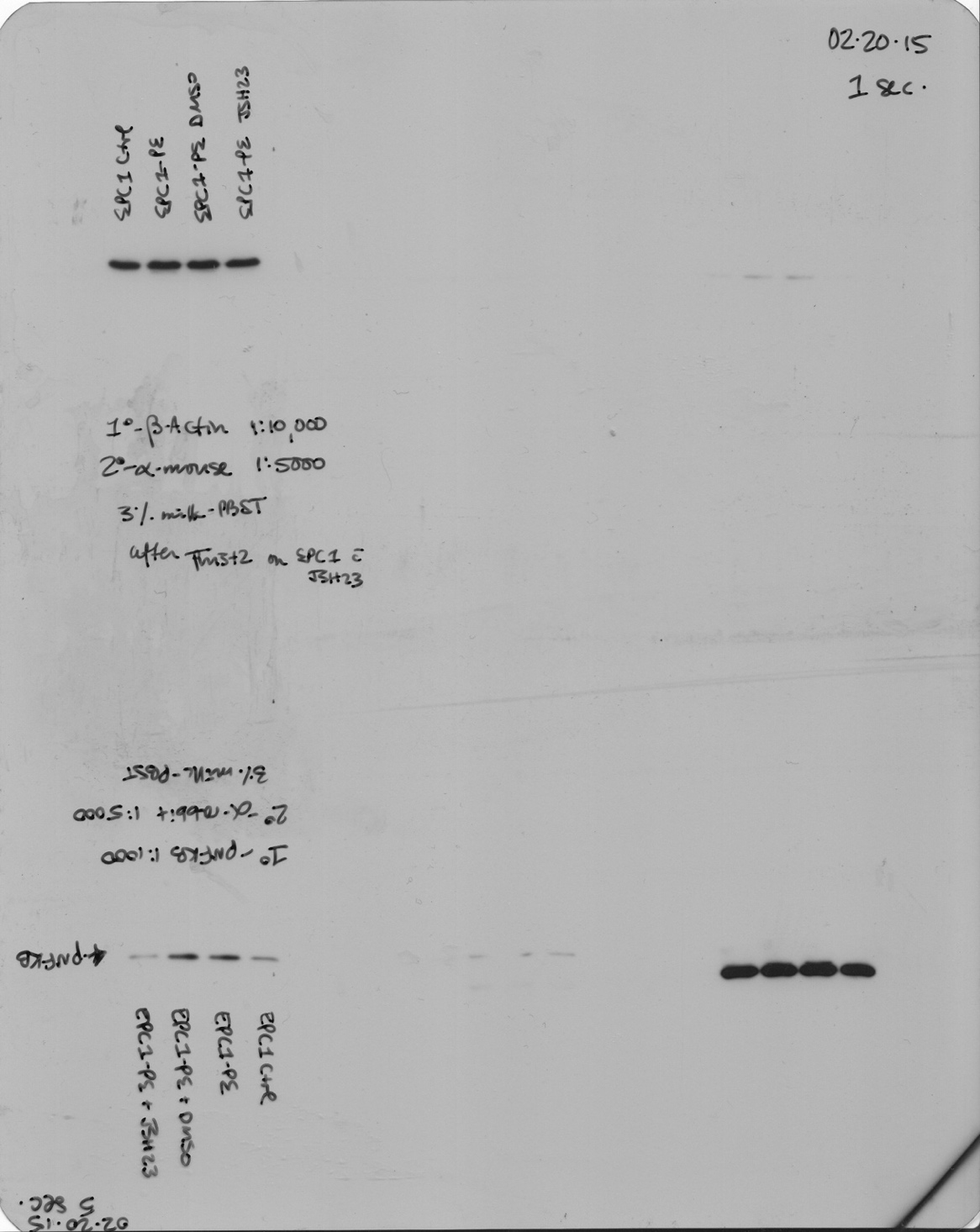
**

**Supplementary Figure S17.** Full-length Western blot of β-Actin in EPC1 cells treated with JSH23, visualized by chemiluminescence. β-Actin was used as a loading control for Twist2, pNFkB, and NFkB. Relevant component of the experiment is marked with a black box. These data are presented in Figure 5a.


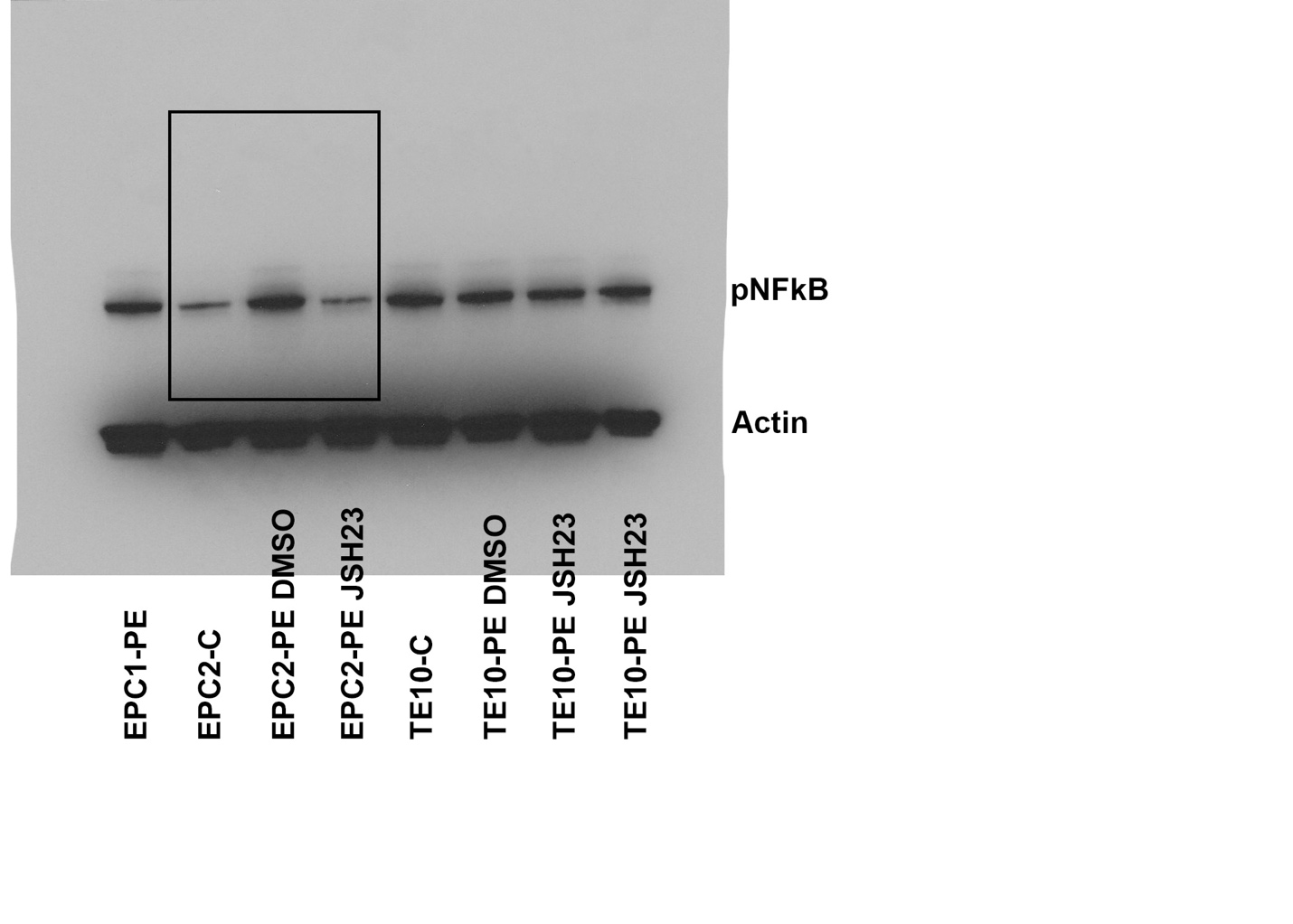


**Supplementary Figure S18.** Full-length Western blot of pNFkB in EPC2 cells treated with JSH23, visualized by fluorescence using the Typhoon FLA 9000 system. Relevant experiment is marked with a black box and lanes have been labeled to match the main text. These data are presented in Figure S1.


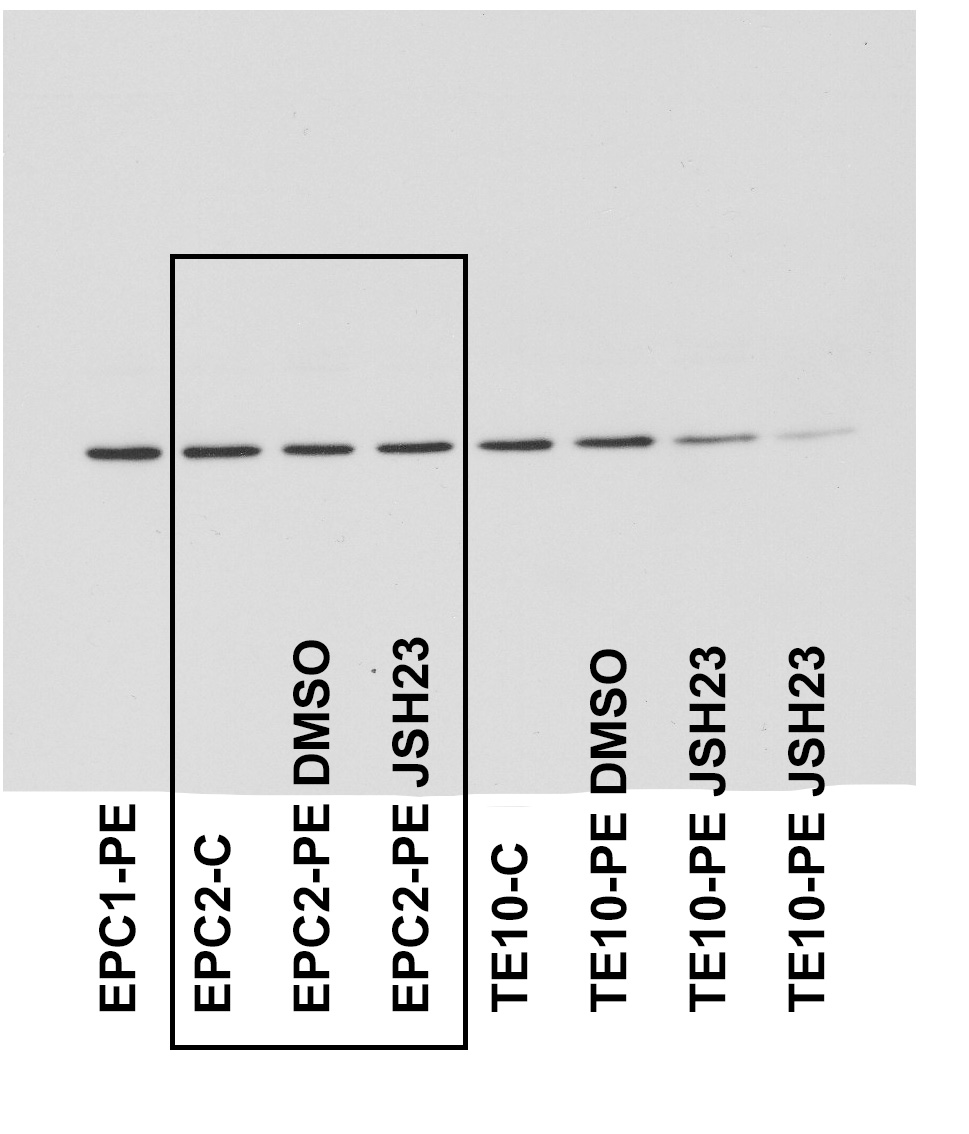


**Supplementary Figure S19.** Full-length Western blot of total NFkB in EPC2 cells treated with JSH23, visualized by fluorescence using the Typhoon FLA 9000 system. Relevant experiment is marked with a black box and lanes have been labeled to match the main text. These data are presented in Figure S1.


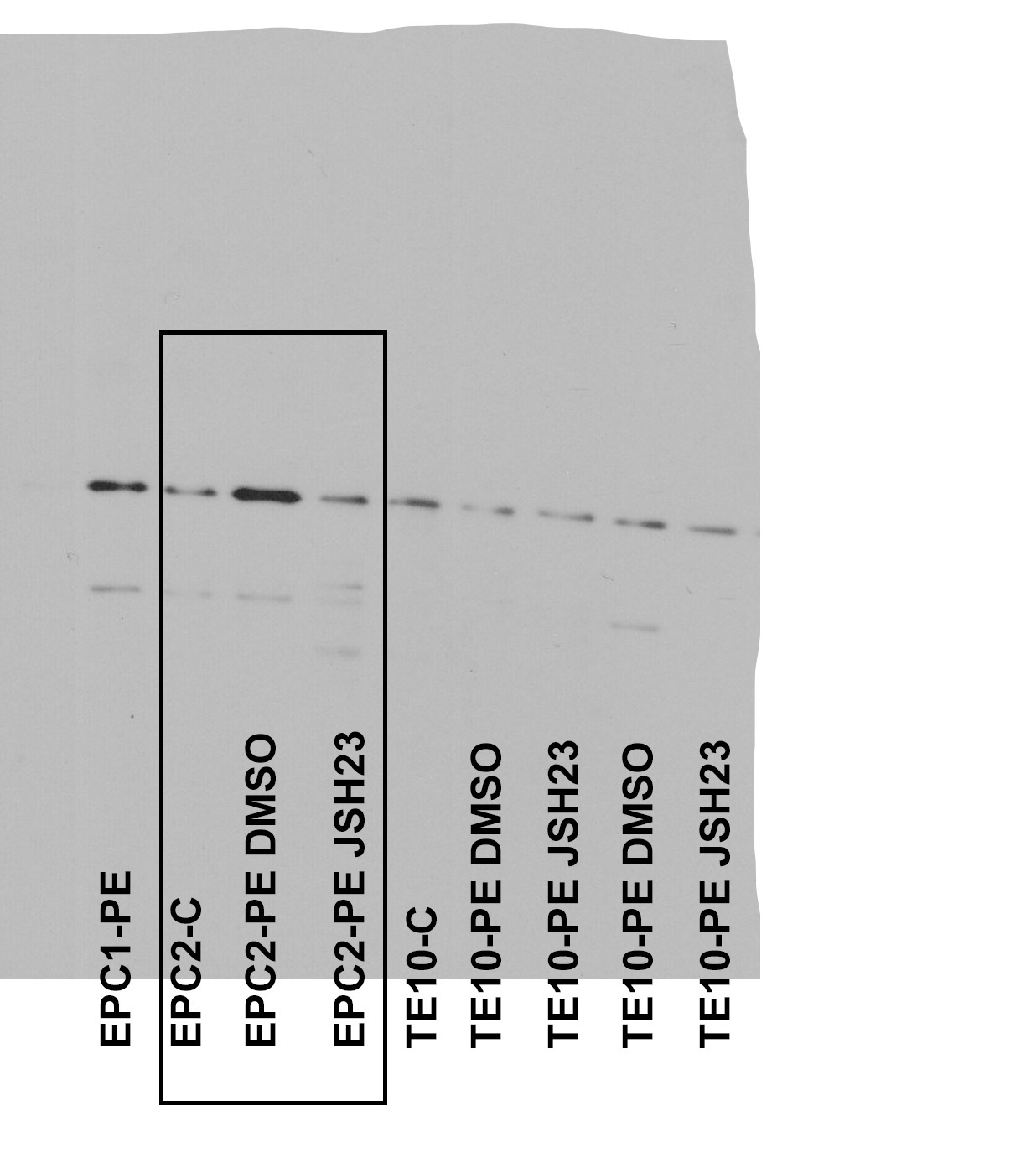


**Supplementary Figure S20.** Full-length Western blot of Twist2 in EPC2 cells treated with JSH23, visualized by fluorescence using the Typhoon FLA 9000 system. Relevant experiment is marked with a black box and lanes have been labeled to match the main text. These data are presented in Figure S1.


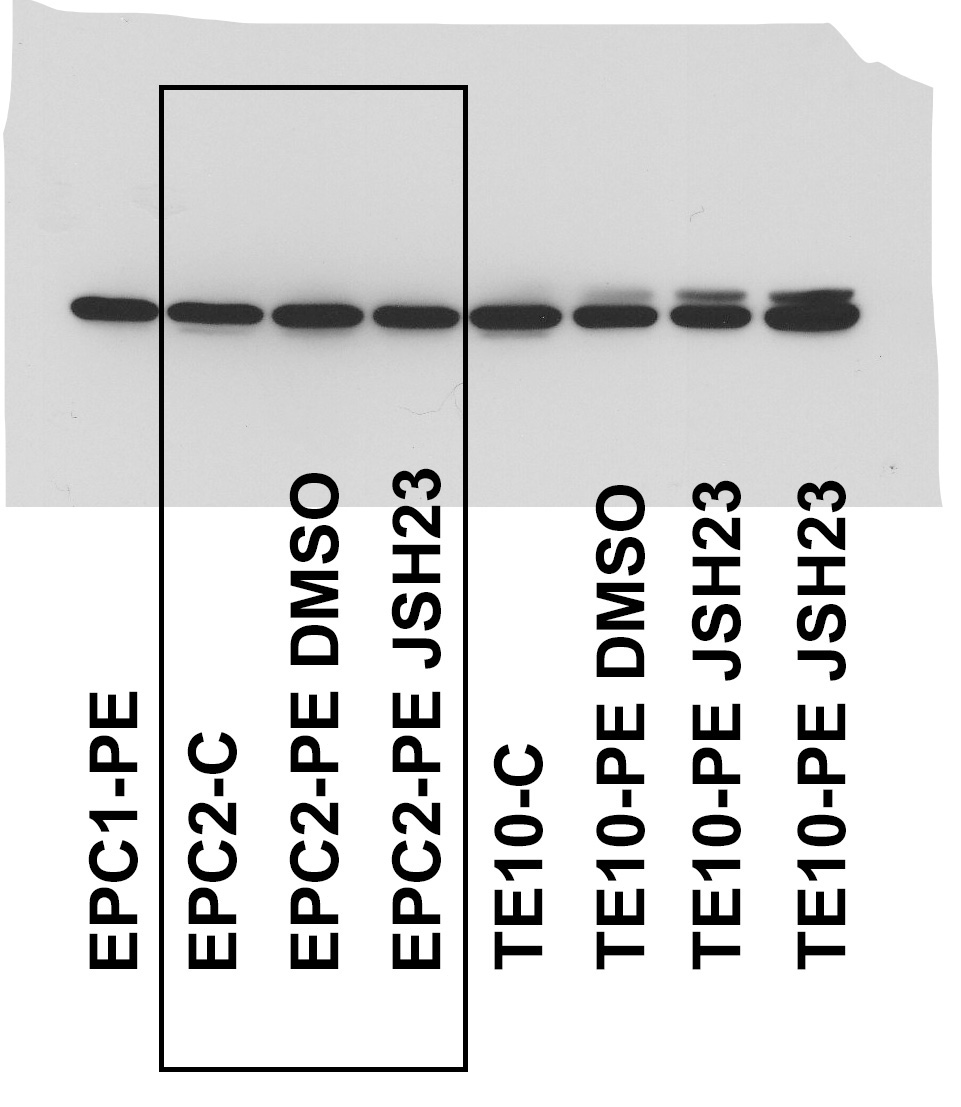


**Supplementary Figure S21.** Full-length Western blot of β-Actin in EPC2 cells treated with JSH23, visualized by fluorescence using the Typhoon FLA 9000 system. β-Actin was used as a loading control for Twist2, pNFkB, and NFkB in EPC2 cells. Relevant experiment is marked with a black box and lanes have been labeled to match the main text. These data are presented in Figure S1.
